# Tracking global changes induced in the CD4 T-cell receptor repertoire by immunization with a complex antigen using short stretches of CDR3 protein sequence

**DOI:** 10.1093/bioinformatics/btu523

**Published:** 2014-08-05

**Authors:** Niclas Thomas, Katharine Best, Mattia Cinelli, Shlomit Reich-Zeliger, Hilah Gal, Eric Shifrut, Asaf Madi, Nir Friedman, John Shawe-Taylor, Benny Chain

**Affiliations:** ^1^UCL CoMPLEX, ^2^UCL Division of Infection and Immunity, London WC1 6BT, UK, ^3^Weizmann Institute of Science, Rehovot 76000, Israel and ^4^UCL Computer Science, London WC1E 6BT, UK

## Abstract

**Motivation:** The clonal theory of adaptive immunity proposes that immunological responses are encoded by increases in the frequency of lymphocytes carrying antigen-specific receptors. In this study, we measure the frequency of different T-cell receptors (TcR) in CD4 + T cell populations of mice immunized with a complex antigen, killed *Mycobacterium tuberculosis*, using high throughput parallel sequencing of the TcR*β* chain. Our initial hypothesis that immunization would induce repertoire convergence proved to be incorrect, and therefore an alternative approach was developed that allows accurate stratification of TcR repertoires and provides novel insights into the nature of CD4 + T-cell receptor recognition.

**Results:** To track the changes induced by immunization within this heterogeneous repertoire, the sequence data were classified by counting the frequency of different clusters of short (3 or 4) continuous stretches of amino acids within the antigen binding complementarity determining region 3 (CDR3) repertoire of different mice. Both unsupervised (hierarchical clustering) and supervised (support vector machine) analyses of these different distributions of sequence clusters differentiated between immunized and unimmunized mice with 100% efficiency. The CD4 + TcR repertoires of mice 5 and 14 days postimmunization were clearly different from that of unimmunized mice but were not distinguishable from each other. However, the repertoires of mice 60 days postimmunization were distinct both from naive mice and the day 5/14 animals. Our results reinforce the remarkable diversity of the TcR repertoire, resulting in many diverse private TcRs contributing to the T-cell response even in genetically identical mice responding to the same antigen. However, specific motifs defined by short stretches of amino acids within the CDR3 region may determine TcR specificity and define a new approach to TcR sequence classification.

**Availability and implementation:** The analysis was implemented in R and Python, and source code can be found in Supplementary Data.

**Contact:** b.chain@ucl.ac.uk

**Supplementary information:** Supplementary data are available at *Bioinformatics* online.

## 1 INTRODUCTION

Adaptive immunity is carried out by populations of B and T lymphocytes, which collectively express a large set of different antigen-specific receptors created during haemopoesis by a unique process of somatic cell gene rearrangements. The clonal theory of immunity (Burnet, 1959) proposes that lymphocytes carrying receptors that specifically bind an antigen to which the immune system is exposed, for example, during infection or vaccination, respond by proliferating and differentiating. This population of expanded and differentiated cells then confer on the system the ability to respond specifically to the antigen to which they had previously been exposed. The clonal theory therefore explains the immune system properties of specificity and memory. A prediction of this theory is that the frequency of lymphocytes that have been exposed to antigen (i.e. memory or effector cells) will be greater than the frequency of those that have not (i.e. naive). This prediction has been verified for T cells in a wide variety of models, using antigen-specific readouts such as cytokine responses, and Major Histocompatibility Complex (MHC) multimer binding to identify expanded lymphocyte clones (Catron *et al.*, 2004; Hataye *et al.*, 2006; Moon *et al.*, 2007). The selective expansion of specific clones has also been inferred from global measurements such as V region usage (Reuther *et al.*, 2013) or spectratyping (a technique sometimes referred to as the immunoscope) (Russi *et al.*, 2013). Previous studies have distinguished between private T-cell receptors (TcRs), found in one or a few individuals, and public TcRs found within the responding repertoire of a majority of individuals. The response to many antigens seems to consist of a mixture of public and private specificities (Cibotti *et al.*, 1994; Clute *et al.*, 2010; Day *et al.*, 2011; Menezes *et al.*, 2007).

The introduction of short read parallel high-throughput sequencing (HTS) provides an alternative approach to measuring lymphocyte receptor frequencies, allowing evaluation of the global receptor repertoire of particular lymphocyte populations. Rearranged receptor genes, or their mRNA products, are expanded and then sequenced directly, and the number of times each unique receptor sequence is found is simply counted. This approach can in principle generate an accurate estimate of the number of times each unique lymphocyte receptor is present in a particular population, and this information should reflect the prior antigen exposure of the individual. Several previous studies have already used HTS to reveal interesting properties of the BcR and TcR repertoire. Freeman *et al.* (2009) and Robins *et al.* (2009) used HTS to show non-uniform V(D)J gene segment usage in humans during recombination, which has been attributed to chromatin conformation (Ndifon *et al.*, 2012). Complementary work on antibody repertoire diversity has also been conducted in the zebrafish, again showing non-uniform V(D)J recombination that is qualitatively conserved between individuals (Weinstein *et al.*, 2009). Weinstein *et al.* (2009) also show that this repertoire is shaped by maturity, with a greater skew in V(D)J usage observed at 2 months compared with 2-week-old individuals. Other studies have used HTS to provide unexpected insight into the naive and memory T-cell compartments, revealing that the memory compartment may be far more diverse than previously thought (Klarenbeek *et al.*, 2010; Robins *et al.*, 2009; Venturi *et al.*, 2011).

A major goal is to use the HTS lymphocyte receptor sequence data to identify antigen-specific changes in the repertoire at a global level. There remain some major challenges, however. First, HTS generates primary sequence data, which cannot be easily mapped onto three-dimensional receptor conformation, much less onto intrinsic antigen specificity. Second, current technologies do not yet provide easy ways to link the two chains of the antigen-specific receptor (heavy and light for antibody, *α* and *β* or *γ* and *δ* for T cells) at a single-cell level. The majority of studies of T-cell repertoires using HTS have focused only on *β* chains. The antigen specificity of the receptor will depend on the pairing of a specific *α* and *β* chain, and therefore cannot be inferred from *β* chains alone.

Despite these limitations, there are a number of indications that local features of protein primary structure may contain hidden information that reflects specific protein–protein interactions occurring at the level of a fully folded tertiary or quaternary structure. One interesting example is the analysis of conserved amino acid pairs within a family of homologous proteins that has recently been used to predict with remarkable accuracy the structure of the fully folded protein on the basis of conserved protein–protein interactions (Schug *et al.*, 2009). Consequently, some success has also been achieved using a primary protein structure to predict antibody–antigen docking (Brenke *et al.*, 2012). From a machine learning perspective, these approaches are reminiscent of algorithms that use local low-level features, such as individual words or image fragments, to produce remarkably efficient classification of complex large datasets, such as sets of documents or images. Surprisingly, good results can be achieved with little regard for the semantic content or meaning of these types of data. We thought that local sequence features could be used to define antigen-specific changes in the TcR repertoire following immunization, reflecting underlying information on the nature of interactions between TcR and peptide-MHC complexes. In this study, we develop an approach based on the well-studied bag-of-words (BOW) (Csurka *et al.*, 2004; Joachims, 1998; Lowe, 1999) algorithm to categorize and classify sets of TcR sequences from immunized and unimmunized mice at different times postimmunization.

Our data highlight the extraordinary diversity of the T-cell repertoire, which result in every individual mouse expressing a majority of receptors that are unique to that individual. Within this ocean of diversity, conventional methods to identify the antigen-specific component of the response by looking for shared expanded clones are problematic. Surprisingly, however, localized sequence features (similar short stretches of adjacent amino acids) can be used to generate a high-dimensional feature space, in which the distinct experimental groups can be readily distinguished with a high degree of accuracy. Short motifs in complementarity determining region 3 (CDR3) primary sequence may therefore play an important role in determining TcR specificity. Our results suggest that the response to antigen may be an emergent property of the repertoire, dominated by clones found only in that individual (private specificities), and distributed over many low-frequency lymphocytes each with different receptors.

## 2 METHODS

Details of immunizations and library preparation are given in the Supplementary Information.

### 2.1 Low-level processing of Illumina sequence reads to generate protein CDR3 sequences

Sequences obtained using this protocol were 55 bp long, spanning the highly diverse CDR3 region. Following the methodology described by Thomas *et al.* (2013), we represent each distinct TcR sequence read in terms of its constituent V and J gene segments, the number of V and J germline nucleotide deletions and the string of nucleotides found between the VJ junction, including any remnants of the D gene segment. Thus, this approach classifies each TcR sequence in terms of five variables, mitigates for sequencing error within V or J regions and determines the correct reading frame to extract the translated CDR3 region.

The short length of the sequences made direct use of the Decombinator (Thomas *et al.*, 2013) problematic for unambiguous assignment of V and J gene segments, as the optimal unique tags that recognize the distinct V and J gene segments are located outside the sequenced window, and are necessarily located far enough from the 3′ and 5′ ends of the V and J gene segments, respectively, to ensure that the deletion of nucleotides from the gene segment ends does not affect their detection. Additionally, the V*β* region located between the primer and the VD junction is similar across all 23 mouse V*β* genes, making creation and detection of unique tags difficult, and the variability in the length of the CDR3 region means the number of J gene nucleotides that are present in each read varies significantly, making selection of a single identifying J keyword difficult. Therefore, some modifications to the Decombinator (Thomas *et al.*, 2013) pipeline were introduced, which are described in detail in the Supplementary Information.

### 2.2 The BOW approach

**Fig. 1.**
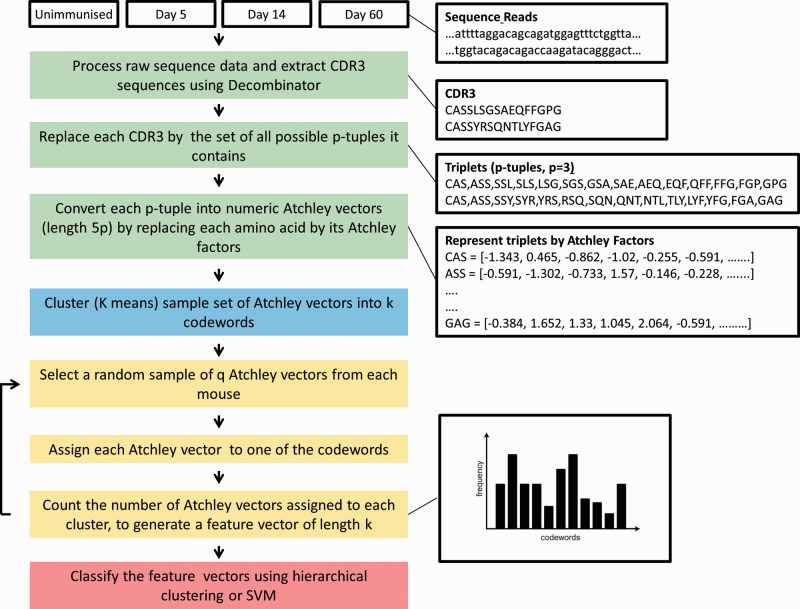
The computational pipeline for classifying TcR repertoires. A schematic of the computational pipeline is shown on the left, and a specific example for two arbitrary TcR *β* sequences is shown on the right (with *p* = 3). CDR3 sequences are preprocessed and represented as a series of p-tuples (contiguous sequences of amino acids of length p). The p-tuples are then converted into numeric vectors of length 5 p by representing each amino acid by its five Atchley factors. The codebook is then generated—a sample of these vectors pooled from all experimental groups is clustered to build a codebook of *k* code words via k-means clustering. A new sample of q p-tuples from each mouse is then selected and mapped to the nearest code word. The number of p-tuples within each code word for that mouse is counted. The sequence data from each mouse are therefore represented by a feature vector of length k, containing the frequency of each code word within the sample. These k length vectors are then analysed by hierarchical clustering or SVM

The basic strategy, used successfully in text, image and also protein sequence classification, is to define a large set of low-level features (code words) within the data, which is variously referred to as a codebook, dictionary or vocabulary. These can be individual words of text, image features or any other simple descriptive features [see e.g. (Csurka *et al.*, 2004; Joachims, 1998; Lowe, 1999)]. Individual data items are then defined by how frequently each code word of the vocabulary is found within that specific piece of data. Each data item is therefore converted into a k-dimensional vector, where k is the size of the vocabulary. Finally, the data is classified using a number of high-dimensional tools, either in a model of two classes (comparing untreated with immunized mice) or four classes (comparing untreated mice with day 5 immunized, day 14 immunized and day 60 immunized). The pipeline is illustrated in Figure 1.

In the specific example examined here, the vocabulary is initially defined as all possible sets of contiguous, short (length *p*, where *p* typically = 3) stretches of amino acids (called p-tuples) within the set of CDR3 regions. These represent the features within the data. The p-tuples are then converted into a numeric vector of length 5 *p* by representing each amino acid by its Atchley factors (Atchley *et al.*, 2005) that represent its physicochemical properties based on polarity, secondary structure, molecular volume, codon diversity and electrostatic charge. To reduce the size of the vocabulary to manageable size *k* (typically 100 ‘code words’), the set of observed vectors is clustered. A sample of Atchley vectors is first generated from a set of sequences selected randomly from all experimental groups. This set is clustered into k clusters using k-means clustering. These k clusters represent the codebook. Once the codebook is defined, the repertoire of sequences from each mouse can be mapped to this codebook. A new set of sequences is selected from each mouse. The CDR3s from this set are converted into p-tuples and then into Atchley vectors of length 5 *p* as described above. Each Atchley vector is allocated to the nearest cluster. Once all vectors are allocated, the number of vectors within each cluster is counted and converted into a proportion of the total number of Atchley vectors selected (q). In this way, each repertoire is mapped into a single k-dimensional vector. The code for p-tuplet extraction and conversion to numerical vectors is given in the Supplementary Information.

These *k*-dimensional feature vectors are then classified using either unsupervised (hierarchical clustering) or supervised learning algorithms. For the latter, we focused on support vector machines (SVM), which seek a linear hyperplane that separates observations from two (or more) distinct classes. [reviewed in Cristianini and Shawe-Taylor (2000)]. We have chosen SVM because it regularizes the weight vector minimizing a combination of its 2-norm with the chosen loss function (in this case, the hinge loss). This ensures that SVM can perform well even when the feature space is high dimensional.

SVM was performed by using the e1071 package in R, and models are trained and tested using leave-one-out cross-validation. Multiclass discrimination is carried out internally in e1071 using a ‘one-against-one’ model. Examples of source code are given in the Supplementary Information.

## 3 RESULTS

### 3.1 Decombinator analysis of sequences from immunized and unimmunized mice

A summary of the analysis of the HTS data by using the modified Decombinator algorithm described in Section 2 is given in Supplementary Table S1.

In total, we analysed 120 million raw sequence reads, of which 19% were classified as specific TcRs by the Decombinator. This proportion is similar to that observed using conventional pairwise alignment as described earlier (Ndifon *et al.*, 2012). The enormous diversity of the repertoire is emphasized by the fact that 76% of the identifiers were unique to a single mouse spleen. The proportion of unique, translated CDR3s was somewhat smaller (62%), reflecting the degeneracy of the genetic code and convergent recombination. A few ‘public’ sequences were shared by all mice (Madi *et al.*, 2014).

We first hypothesized that the repertoire of immunized mice might contain several identical expanded antigen-specific clones, and might therefore be more similar to each other than unimmunized mice. We estimated the similarity between mice using the Jaccard index, the ratio of the intersection to the union of the set of unique sequences in each pair of samples.

**Fig. 2.**
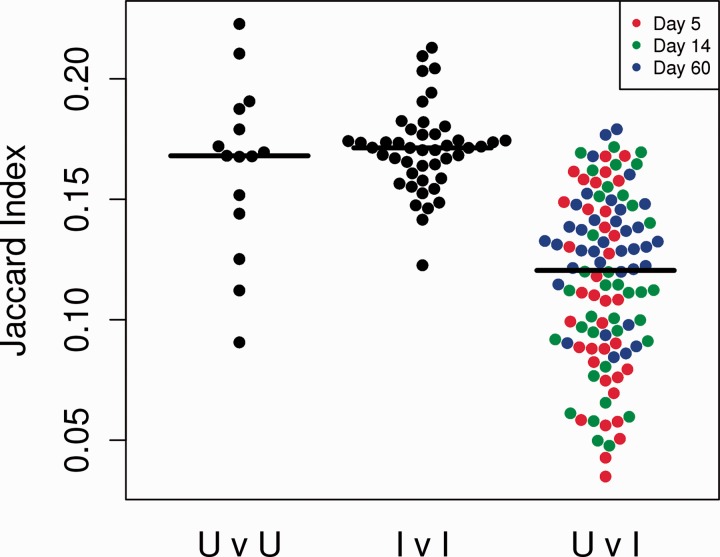
The similarity (Jaccard) index comparing all pairs of mice. Each dot represents the Jaccard index comparing all CDR3 sequences from two mice. CDR3 repertoires from pairs of untreated (U) mice, or pairs of immunized (I) mice, display greater similarity (i.e. have a larger Jaccard index) than repertoires from pairs of mice where one mouse is immunized and one is not immunized. Horizontal black lines indicate mean of each population

The distribution of Jaccard indices for all possible pairs of mice is shown in Figure 2. We carried out these analyses both by using the complete sequence sets for each mouse, and also by subsampling equal numbers of sequences from each mouse, so as to avoid any bias imposed by sample size. Both approaches gave the same qualitative results, but only the former is shown. Contrary to our prediction, this analysis did not demonstrate any greater similarity between pairs of immunized mice than between pairs of unimmunized mice. However, the Jaccard index for pairs composed of one immunized and one unimmunized mouse was significantly smaller than for pairs of two immunized or two unimmunized mice (Fig. 2). Immunization therefore altered the repertoire state but did not drive repertoire convergence.

**Fig. 3.**
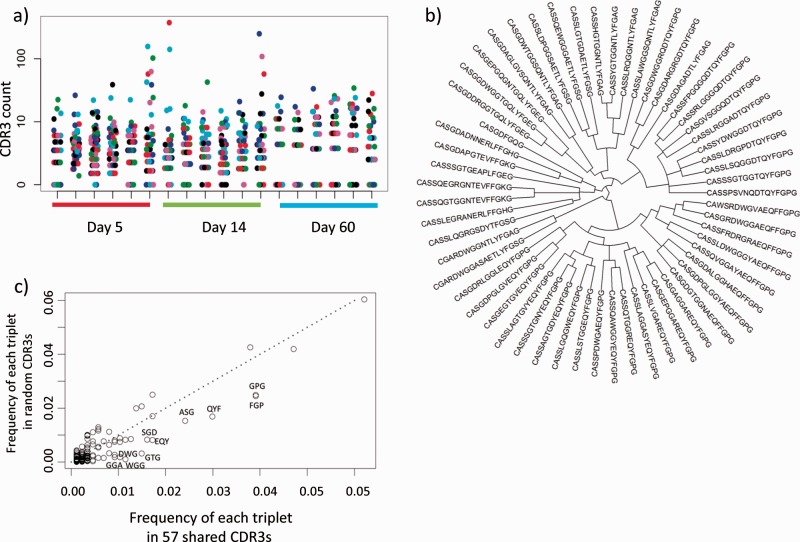
CDR3 sequences shared between immunized mice. (**a**) The frequency (counts per million) of 57 CDR3s that are present in 75% of the immunized mice, but absent from all unimmunized mice (not shown). Each column represents one mouse, grouped according to time after immunization as shown below the *x* axis. (**b**) The amino acid sequences of all 57 CDR3s, clustered according to Levenstein distance. (**c**) A plot of the frequency of each individual amino acid triplet (i.e. sequence of three consecutive amino acids, see Fig. 1) encoded by the 57 CDR3s, measured within the 57 CDR3s themselves (*x* axis) versus the frequency of the same triplets within a random sample of 1000 sets of 57 CDR3s selected from the set of CDR3s from all immunized mice (*y* axis). The diagonal line designates an equal frequency in the shared CDR3s and in the random set. Those triplets that are overrepresented in the shared CDR3s are found in the lower right area of the plot

We looked next in more detail for individual CDR3s shared between immunized mice. No CDR3s were present in all immunized mice but absent from all unimmunized mice. However, 57 CDR3 were present in 75% of immunized mice but absent from all unimmunized mice. In general, these CDR3s were present at low frequencies (Fig. 3a) although a few CDR3s were amplified in individual mice. Inspection of the CDR3 sequences (Fig. 3b) suggested that the CDR3 sequences clustered into families, defined by shared short amino acid sequence motifs. To capture this impression quantitatively, the frequency of each amino acid triplet (sequence of three consecutive amino acids) within the 57 CDR3s was compared with their frequency in a large sample of random CDR3s (Fig. 3c). A number of triplets were over-represented in the shared CDR3 set, suggesting they reflected functional similarity between related sets of CDR3s. We therefore investigated in a more systematic way whether CDR3s from immunized mice shared primary protein sequence features that distinguished them from unimmunized mice. For this purpose, we adapted the BOW approach (also called the n-gram kernel) originally developed in the context of document recognition (Joachims, 1998), together with a clustering step to reduce the dimensionality of the vocabulary. Details of the method are given above. The codebook used for classification was initially chosen arbitrarily to be 100 clusters each containing a subset of contiguous, short (length *p*, where *p* typically = 3) stretches of amino acids, from the set of contiguous p-tuples found within the CDR3 dataset. The similarity metric for clustering was based on individual amino acid Atchley factors, reflecting similarities in physicochemical characteristics of the amino acids. The contents of each cluster are given in Supplementary Table S1 (SI), and the sizes of the 100 clusters are shown in Supplementary Figure S1.

**Fig. 4.**
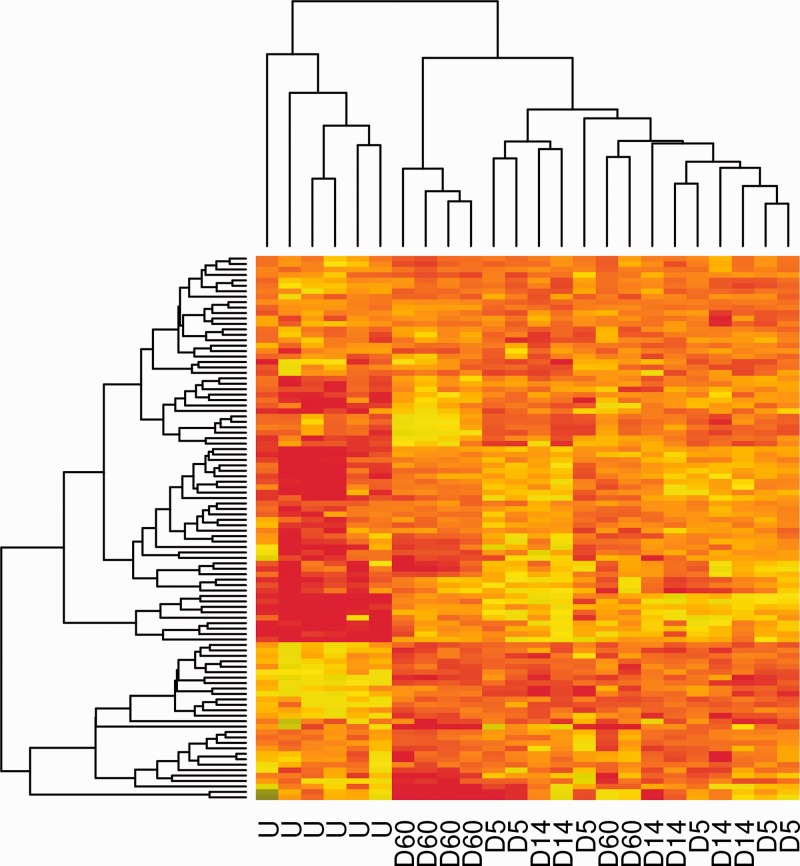
Hierarchical clustering distinguishes between the code word (clusters of triplets) distribution profiles of unimmunized and immunized mice. Each mouse was categorized as described in the text, using *k* = 100, *p* = 3 (triplets), *q* = 10 000. The heatmap shows the relative proportion of sequences within each code word (rows) for each mouse (columns). A small group of codewords appeared more frequently in untreated mice compared with immunized (bottom left corner of heatmap), while conversely a larger group of codewords appear more frequently in immunized mice (top right). The data are clustered along both axes using Euclidean distances and complete linkage method in the R function ‘hclust’

Initially, we sampled 10 000 randomly selected amino acid triplets (i.e. p = 3) from the CDR3 region of each mouse, allocated each triplet to its cluster within the codebook and then counted the total number of triplets within each cluster for that mouse. The set of primary sequences for each mouse was therefore mapped into a numeric feature vector of length 100. The results for all 24 mice are displayed in Figure 4, ordered by hierarchical clustering along both dimensions. Unsupervised clustering correctly separates all six unimmunized mice (on the left) from the immunized mice. Some additional structure is evident, with most (4 of 6) of the day 60 mice clustering between unimmunized and day 5/14 mice. Several different patterns of code word distribution are observed. For example, code words at the top of the heatmap become more highly represented in the repertoire of immunized mice, while code words at the bottom are less represented.

**Fig. 5.**
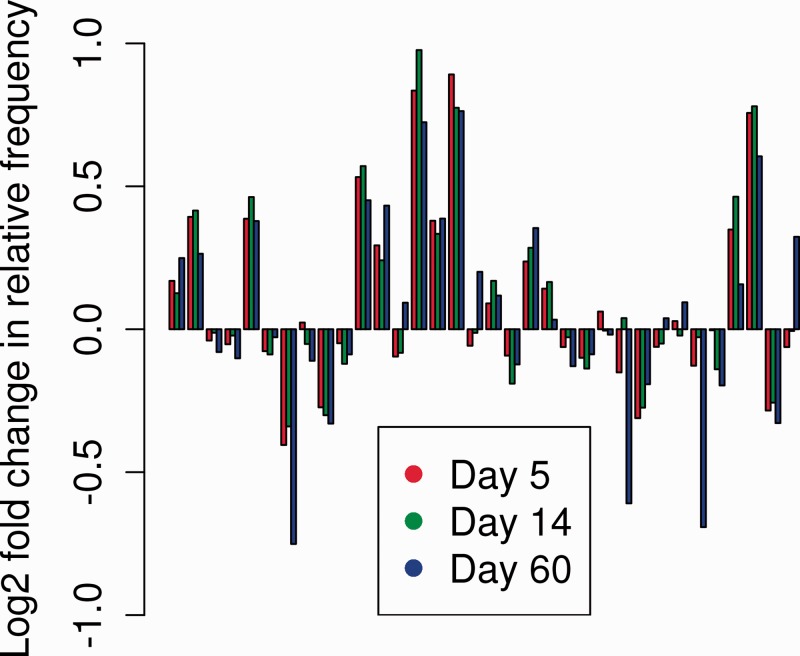
Differences between code word (clusters of triplets) distribution profiles between unimmunized and immunized mice. Each mouse was categorized as described in text, using *k* = 100, *p* = 3 (triplets), *q* = 10 000. The relative frequency of each code word cluster for each group of six mice is averaged and shown relative to the average in the unimmunized group for the corresponding code word. For clarity, the data for only the first 34 code words are shown

A summary of the data, in which code word cluster sizes from all mice in each group are averaged and plotted as log ratio relative to unimmunized mice, is shown in Figure 5. A number of code words are over- or under-represented in the immunized mice, reflecting changes in the frequency of sets of amino acid triplets following exposure to antigen. These code word frequency profiles were used to develop a multiclass SVM with which we could explore the parameters of the BOW algorithm.

Mice were classified as belonging to one of four classes: unimmunized/control, day 5, day 14 and day 60 postimmunization. Training and testing was performed using leave-one-out cross-validation on each mouse in turn.

The results of varying several of the parameters of the classification algorithms are shown in Table 1. Using a radial basis function for the SVM had little effect on classification efficiency, probably reflecting the inherent high dimensionality of the data. All further analysis was therefore carried out using linear SVM kernels. Decreasing the codebook size to 10 code words compromised the success rate, as did decreasing the number of p-tuples sampled from 10 000 to 1000. Increasing the sample size above 10 000 had no further effect (not shown). Increasing p from 3 to 4 (i.e. quadruplets, rather than triplets) made little difference, although it was difficult to know whether this was because efficiency was already close to 100% or the additional information carried in the longer amino acid stretches was not informative. Interestingly, decreasing p to one (i.e. simply amino acid prevalence) retained some discriminative potential, albeit considerably reduced from *p* = 3 or 4.

**Table 1.** Efficient SVM classification using bag-of-words categorization

*p*	*q*	*k*	Kernel	False positive	False negative
3	1000	100	Linear	0.12	0.04
3	1000	100	RBF	0.1	0.02
3	10 000	100	Linear	0	<10^−3^
3	10 000	100	RBF	0	<10^−3^
3	1000	10	Linear	0.42	0.11
4	1000	100	Linear	0.04	0.01
4	10 000	100	Linear	0	0
1	1000	N/A	Linear	0.3	0.08
1	10 000	N/A	Linear	0.02	0.002

*Note*. Mice were classified as either immunized or unimmunized. False-positive rate is the proportion of unimmunized mice classified as immunized. False-negative rate is the proportion of immunized mice classified as unimmunized. *k* is number of code words (clusters of Atchley vectors). *q* is number of p-tuples sampled from each mouse. *p* is the length of contiguous amino acid sequence. SVM uses either a linear or radial basis function (RBF) kernel.

**Fig. 6.**
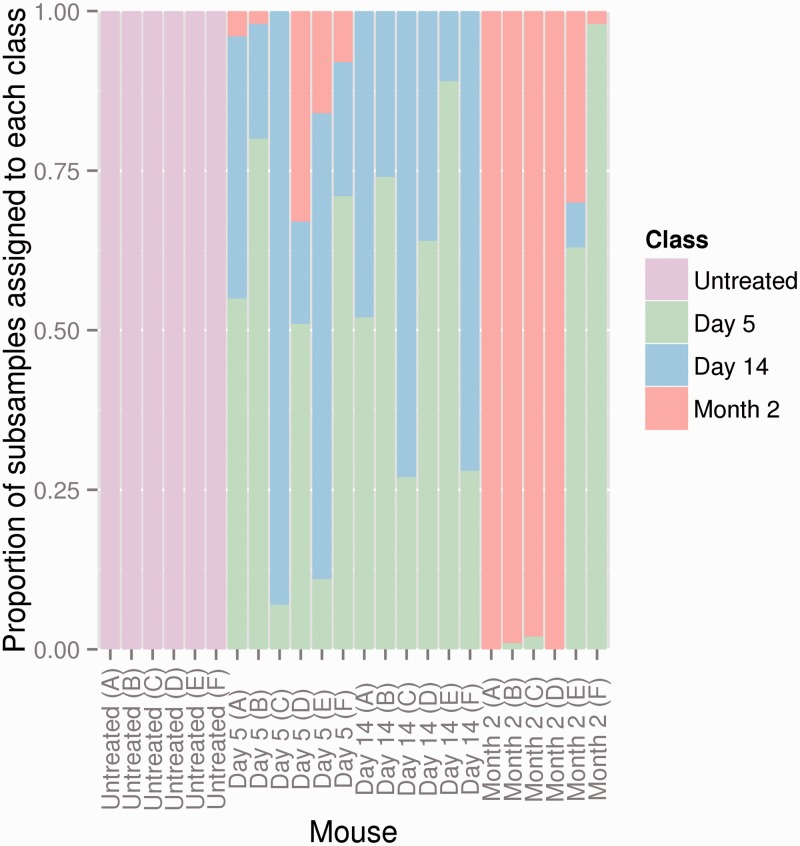
SVM can efficiently classify time-dependent changes in CDR3 repertoire following immunization. A subsample of *q* = 10 000 triplets (*p* = 3) was taken from each mouse to generate a frequency distribution over the code words and train and test a leave-one-out linear SVM. This was repeated 100 times, and the proportion of these repetitions classified as each of the four classes is shown

We next examined classification efficiency retaining the separate time points postimmunization as distinct classes (Fig. 6). For this purpose, we used *k* = 100, *p* = 3, and *q* = 10, 000. A subsample of *q* = 10 000 triplets (*p* = 3) was taken from each mouse to generate a frequency distribution over the code words and train and test a leave-one-out linear SVM. This subsampling was repeated 100 times for each mouse, and the proportion of each of these 100 repetitions classified as each of the four classes is shown in Figure 6. Similar results were obtained for *p* = 4. As discussed previously, immunized and unimmunized mice are distinguished with 100% efficiency. As we expected, the repertoire of day 5 and day 14 mice cannot be efficiently distinguished from each other, reflecting the fact that the T-cell response at these two time points is likely to be similar. Interestingly, the repertoire of day 60 mice was often distinct from that of the earlier time points, although never returning to an unimmunized type. Thus, the T-cell repertoire appears to show a time-dependent change following immunization. The efficiency with which day 60 and earlier time points could be distinguished varied between mice, suggesting that the time course of this evolution was somewhat variable between individuals.

We wondered whether the distinctive features of the immunized repertoire were determined predominantly by a few abundant T cells (i.e. highly expanded clones), or whether the repertoire was determined by many rare T cells sharing similar CDR3 sequence features. We therefore first ranked all the TcRs from each mouse according to the number of times it occurred in the sample, and then selected only CDR3 sequences from high frequency (top 10 percentile in clone frequency) or low frequency clones (bottom 10 percentile) and repeated the analysis shown in Figure 6. Remarkably, equivalent classification efficiency was obtained using either high or low frequency clones. In all, 100% correct classification was retained, even when the cutoff was reduced to the lowest 1%. A potential confounder of this analysis is sequencing error, which can result in contamination of low frequency clones with rare ‘variants’ of high frequency clones that arise purely during *in vitro* sample processing. To try and mitigate this potential confounder, we reanalysed the dataset using the protocol published in Ndifon *et al.* (2012). In this protocol, additional steps are included in which low frequency clones are first clustered with high frequency clones, to correct for sequencing errors. The reanalysis of these data gives the same pattern of results. Increasing the cut-off to use only those clones in the top 1 percentile (on average, these appeared 40 times or more in a sample) increased the false-positive rate to 16%. These results imply that the enrichment of specific sequence features in immunized mice reflects changes to many different T-cell clones of both high and low frequency, rather than the dominant expansion of a few dominating antigen-specific clones.

Although our analysis focused on CDR3s, because these regions are known to interact with antigen peptides within the antigen-binding cleft of MHC molecules, we also analysed V and J usage (see Supplementary Fig. S2). Reproducible differences were observed in the proportion of a number of specific V regions but not J regions. The reasons for these differences were not investigated further but could reflect the presence of superantigen-like structures within *M. tuberculosis* (Ohmen *et al.*, 1994).

## 4 DISCUSSION

The computational pipeline presented above analyses the global T-cell immune response to a complex antigen, killed *M. tuberculosis*. This antigen contains many different proteins and a large number of possible T-cell epitopes. Despite this complexity, the results demonstrate a coherent but highly distributed set of responses, emerging from the background of the remarkable diversity and plasticity of the TcR-generating system.

HTS of the T-cell receptor repertoire of individual mice emphasized the size of the potential repertoire, consistent with previous reports (Ndifon *et al.*, 2012). To simplify the computational aspects discussed in more detail below, we focus here exclusively on the CDR3 regions of the receptor, which are believed to contribute most to the interaction between TcR and the antigenic target peptide lying within the MHC groove (Garcia and Adams, 2005; Rudolph *et al.*, 2006). The heterogeneity is highlighted by the observation that over 60% of the CDR3 repertoire is made up of unique sequences, and only a small proportion are shared by all mice, even though all mice are derived from a well-established in-bred strain, and are therefore genetically similar. On the basis of the Jaccard index, this diversity extends equally to unimmunized and immunized mice. Thus, immunization, at least in this example, does not seem to result in the emergence of a large pool of shared identical CDR3 sequences. In contrast, the Jaccard index when comparing immunized and non-immunized mice is significantly lower than that obtained by comparing within either immunized or unimmunized groups. This suggests a model in which one heterogeneous population of receptors changes as a result of immunization to another equally heterogeneous, but nevertheless distinct, population. This picture of an immune response made up of frequency changes in many heterogeneous clones was confirmed by the further investigations detailed below.

As we were unable to determine a clearly defined set of identical receptors that correlated with antigen response, we devised a strategy to extract features that would reflect the similarities and differences between different datasets. As a first step, we adopted the simplest consecutive string kernel algorithm, the BOW method (Joachims, 1998; Lodhi *et al.*, 2002). To restrict the size of the feature space (there are 8000 possible triplet amino acid sequences, and 160 000 quadruplets), we clustered the set of k-tuples into a 100-word codebook of similar k-tuples. The codebook was based on the set of k-tuples observed in our data, but as further datasets become available it should be possible to design a universal codebook applicable to all datasets. The k-tuples were classified as similar by transforming the amino acid sequences into numerical vectors, using Atchley factors (Atchley *et al.*, 2005). Several alternative classification schemes have been devised [e.g.(Kidera *et al.*, 1985)] and have previously been used to describe TcR properties (Epstein *et al.*, 2014). It will be of interest to see how these classification schemes compare in the sequence classification problems investigated here.

Despite its simplicity, the feature space constructed from short consecutive amino acid p-tuples revealed a remarkably consistent time-dependent response to immunization. Thus, while there was little sharing of identical sequences between groups of mice, shared patterns of sequences, defined by a particular distribution of p-tuples, were easily observed using both supervised and unsupervised classification methods. Although a few code words (i.e. clusters of amino acid p-tuples) showed large differences between experimental groups, a substantial proportion of the code words showed smaller but consistent changes. This suggested a large number of TcRs contribute to the antigen-driven changes in the composition of the repertoire, perhaps reflecting the complex nature of the antigen used in these studies. Efficient recognition of different experimental groups required analysis of large numbers (q≥10000) of code words. Furthermore, the TcRs that defined the antigen-specific repertoire were not confined to high frequency clones but also included low frequency clones. It should be noted that even ‘low frequency’ receptors may represent amplified clones, as sample size limits the lowest observable TcR frequency we can reliably see. Furthermore, a proportion of low frequency sequences are likely to be derived from sequencing errors of high frequency TcRs. Nevertheless, the data suggest a model where recognition of M. tb in these mice is distributed among many low and high frequency clones, sharing characteristic amino acid triples or quadruplets. At a molecular level, one might envisage that these selected subsequences may be directly interacting with specific features of antigenic processed peptides exposed at the surface of the MHC binding groove (Garcia and Adams, 2005). In fact, a number of distinct TcRs are likely to interact with a single peptide/MHC complex, with a spectrum of different affinities (Birnbaum *et al.*, 2012). In such a model, although the overall recognition between TcR and MHC/peptide is mediated at the level of tertiary or quaternary structure, and therefore not reducible to linear sequence features, the interaction between CDR3 and a specific peptide/MHC may impose constraints that are observable at the level of short contiguous amino acid sequences. Such an approach using the interactions of neighbouring amino acids has been successfully used earlier in the context of antibodies (Mora *et al.*, 2010). Similar constraints have been demonstrated to characterize conserved protein–protein interactions (Schug *et al.*, 2009) in large evolutionary-related protein families.

The majority of previous studies have measured individual T-cell antigen-specific responses without reference to TcR sequence (e.g. using MHC multimer binding or cytokine responses). More global approaches to detecting and quantifying receptor diversity have used spectratyping to obtain a profile of CDR3 lengths or flow cytometry to quantify V region usage (Ciupe *et al.*, 2013; Faint *et al.*, 1999; Pannetier *et al.*, 1995). Both techniques have given interesting insights into clonal expansions associated with a variety of antigen-driven responses, although the sensitivity limits the detection of small clones. Spectratyping can be extended to give sequencing data, but this is a laborious and low-throughput process. We predict that, as larger sequence datasets become available from HTS approaches, the extent of diversity in the antigen-driven TcR repertoire response will increase dramatically. This study is confined to a single antigen, in a single inbred strain of mice. Additional studies are in progress to extend the datasets to better defined model antigen systems, for example, focusing on one individual MHC/peptide response. Preliminary results suggest the response to such weaker and narrower antigen stimuli are more subtle, and will require more sophisticated analysis. Many extensions of the current approach are possible. For example, the feature space can be extended, by including V and J region information, positional information in the context of the p-tuple within the CDR3 and the inclusion of non-continuous string kernels.

The results described above offer an intriguing insight into the nature of an immune response. On the one hand, the success of classification methods using fairly simple low-level features of protein sequence offer hopeful indications for applying this sort of approach to analysis of clinical samples for the prognosis, diagnosis or stratification of patients in the context of both infectious and non-infectious (e.g. cancer, autoimmunity or transplantation) disease. On the other hand, if further studies generalize our observation of a ‘distributed’ immune response, in which a response is carried by large numbers of different low frequency clones with shared features, this will pose some formidable computational challenges. Robust experimental pipelines, improved HTS technology and application of the latest advances in machine learning will all be required, but such combinations are likely to provide new insights into the function of the adaptive immune system, and ultimately translational benefits in the clinical context.

*Funding*: Engineering and Physical Sciences Research Council UK, the Medical Research Council UK, Microsoft Research, and Weizmann UK.

*Conflict of interest*: none declared.

## Supplementary Material

Supplementary Data
