# Supplementary material for: Tracking global changes induced in the CD4 T-cell receptor repertoire by immunization with a complex antigen using short stretches of CDR3 protein sequence
Source: Bioinformatics. 2014 Aug 5;30(22):3181–8. doi: 10.1093/bioinformatics/btu523 (PMC4221123; doi:10.1093/bioinformatics/btu523)
Supplement: Supplementary Data [file supp_30_22_3181__index.html]

Tracking global changes induced in the CD4 T cell receptor repertoire by immunization with a complex antigen using short stretches of CDR3 protein sequence — Tracking global changes induced in the CD4 T-cell receptor repertoire by immunization with a complex antigen using short stretches of CDR3 protein sequence — Tracking global changes induced in the CD4 T-cell receptor repertoire by immunization with a complex antigen using short stretches of CDR3 protein sequence — Supplementary Data 

# Tracking global changes induced in the CD4 T-cell receptor repertoire by immunization with a complex antigen using short stretches of CDR3 protein sequence

## Supplementary Data

files

**Files in this Data Supplement:**

- Supplementary Data - pdf file
