# Supplementary material for: Tracking global changes induced in the CD4 T-cell receptor repertoire by immunization with a complex antigen using short stretches of CDR3 protein sequence
Source: Bioinformatics. 2014 Aug 5;30(22):3181–8. doi: 10.1093/bioinformatics/btu523 (PMC4221123; doi:10.1093/bioinformatics/btu523)
Supplement: Supplementary Data [file supp_btu523_supplementary-information.pdf]

Supplementary Information

July 25, 2014

T Cell Receptor Library Construction

Briefly, C57BL/6 mice were immunised with freeze-dried Mycobacterium tuberculosis H37RA in a water/oil emulsion (Complete Freunds Adjuvant). Groups of six mice were sacrificed at day 5, day 14 and day 60, and spleens were harvested and processed for mRNA extraction. Ovalbumin (100 ug/mouse) was included in some immunisations, but the presence or absence of ovalbumin had no effect on the analysis reported below and is not considered further in this study. Six further mice were left unimmunised. Subsequently, mRNA from 2 million purified CD4+ T cells was reverse transcribed, amplified and sequenced as described in detail in Ndifon, W. et al. (2012). Chromatin conformation governs T cell receptor j gene segment usage. Proceedings of the National Academy of Sciences of the United States of America. PMID: 22984176.. Briefly, reverse transcription was carried out using a $C\beta$-specific primer linked to an *Illumina* 3' sequencing adapter. The resulting cDNA product was amplified with a multiplex PCR using a set of 23 $V\beta$-specific primers. Each $V\beta$-specific primer was anchored to a restriction site sequence for a restriction enzyme (AcuI) that was used to cleave part of the primer sequence, to ensure good coverage of the hypervariable CDR3 with a single short Illumina read. This was followed by ligation of a Illumina 5' adapter, which was linked to a 3-bp barcode sequence at its 3' end, and a second round of PCR amplification using primers for the 5' and 3' Illumina adapters. Final PCR products were gel purified and sequenced using the Genome Analyzer II. The raw sequence files are available from the Short Read Archive (NCBI) under Accession Number SRP042610.

Modifications to Decombinator: Identification of Constituent V and J Gene Segments in Sequence Reads

Instead of a single keyword identifying each V or J gene as in Decombinator, we used a set of keywords for each gene comprising all subsequences of at least 4 nucleotides in length from the $3'$ end (post-primer) of a V gene or the $5'$ end of a J gene. A keyword trie was then built for each V and J gene, and each sequence read was searched, using the Aho-Corasick algorithm for instances of keywords from any of these tries.

To identify which V and J genes are contained within a sequence read, the instances of keywords in the read were processed using 4 parameters: match threshold (MT), match differential (MD), score threshold (ST) and score differential (SD).

A multi-stage approach was used, with the following steps followed for both V and J gene identification:

1. The longest V (J) keyword match from all the identified keywords is considered. If this keyword is at least MT nucleotides long and the longest keyword from a different V (J) gene is at least MD nucleotides shorter, then we assign the V (J) gene to the sequence read that the longest keyword match is from.

2. If step 1 does not identify a V (J) gene, then each V (J) gene is assigned a score reflecting how many keywords from that gene are found in the sequence read. The score for region V_i (J_i) is given as $\sum_{all} xe^{length(x)}$ where x is any keyword from region V_i (J_i) occurring in the sequence read and $length(x)$ refers to the number of nucleotides in the matched keyword.

   The V (J) gene with the maximum score is considered. If this score is lower than ST, we conclude that there is not enough similarity between the sequence read and any V (J) gene to be able to identify a gene, and as such the sequence read is discarded.

If the maximum V (J) score is both greater than ST and more than SD times greater than the next highest score, we assign the V (J) gene with the maximum score to the sequence read.

3. If step 2 does not identify a V (J) gene, but the maximum score is greater that ST, we move to a pairwise alignment method, with equal penalties for opening and extending gaps. The sequence read is assigned to the V (J) gene with the highest pairwise alignment score.

After identification of V and J genes within a sequence read the standard Decombinator method for identifying the number of deletions and the inserted nucleotides is used to give the usual set of five-part identifiers (V gene, J gene, V deletions, J deletions, inserted nucleotides) for each of the raw reads, ultimately allowing extraction of the TcR hypervariable CDR3 region, bounded by the most 5' conserved cysteine in the V gene segment and the conserved FG(X)G motif in the J gene segment, where X may be any of the 20 amino acids.

Source Code

The pipeline consists of two scripts, once Decombinator has been used to extract the CDR3 sequences from the raw sequence reads. The first script is a Python script that takes the CDR3 sequences as input, and outputs a sample set of n amino acid triplets represented in terms of their Atchley factor values. These files are then input into the award-winning SVM `R` package $e1071$.

```
## SCRIPT 1

import os,random
import string
import time
import numpy as np
import math

###################

# Input
q = 10000 ## EDIT - number of feature vectors per histogram
p = 3
howmany = 100 ## EDIT - number of histograms per sample

classtime = "_m2_6" ## EDIT - this is what you want output to be called - see outfile variable

## EDIT - this is the input file of CDR3s to be analysed
filename = "/home/path/to/files/cdr3file.txt"

## EDIT - this is where output file (containing frequency distributions)
pathout = "/home/path/to/files/"
codefile = "codewords"
codewords = np.loadtxt(pathout+codefile+'.txt', delimiter=',')
outfile = open(pathout+'results'+classtime+'.txt',"w")

def atchley_factor( x ):
    import collections as coll

    m = len(x)
    lookup = [ [ -0.591, -1.302, -0.733,  1.570, -0.146 ],
               [ -1.343,  0.465, -0.862, -1.020, -0.255 ],
               [  1.050,  0.302, -3.656, -0.259, -3.242 ],
               [  1.357, -1.453,  1.477,  0.113, -0.837 ],
               [ -1.006, -0.590,  1.891, -0.397,  0.412 ],
               [ -0.384,  1.652,  1.330,  1.045,  2.064 ],
               [  0.336, -0.417, -1.673, -1.474, -0.078 ],
               [ -1.239, -0.547,  2.131,  0.393,  0.816 ],
               [  1.831, -0.561,  0.533, -0.277,  1.648 ],
               [ -1.019, -0.987, -1.505,  1.266, -0.912 ],
               [ -0.663, -1.524,  2.219, -1.005,  1.212 ],
               [  0.945,  0.828,  1.299, -0.169,  0.933 ],
               [  0.189,  2.081, -1.628,  0.421, -1.392 ],
               [  0.931, -0.179, -3.005, -0.503, -1.853 ],
               [  1.538, -0.055,  1.502,  0.440,  2.897 ],
               [ -0.228,  1.399, -4.760,  0.670, -2.647 ],
               [ -0.032,  0.326,  2.213,  0.908,  1.313 ],
               [ -1.337, -0.279, -0.544,  1.242, -1.262 ],
               [ -0.595,  0.009,  0.672, -2.128, -0.184 ],
               [  0.260,  0.830,  3.097, -0.838,  1.512 ] ]

    aa = coll.defaultdict(int)
    aa['A'] = 0; aa['C'] = 1; aa['D'] = 2; aa['E'] = 3
    aa['F'] = 4; aa['G'] = 5; aa['H'] = 6; aa['I'] = 7
    aa['K'] = 8; aa['L'] = 9; aa['M'] = 10; aa['N'] = 11
    aa['P'] = 12; aa['Q'] = 13; aa['R'] = 14; aa['S'] = 15
    aa['T'] = 16; aa['V'] = 17; aa['W'] = 18; aa['Y'] = 19

    xsplit = list(x)
    xfactors = [0] * (5*m)
    for i in range(m):
        for j in range(5):
            xfactors[5*i+j] = lookup[aa[xsplit[i]]][j]

    return xfactors

####################################

count = 0
counter = 0
numvects = 0
t0 = time.time()
```

```
histocount = [0]*len(codewords)
print 'Mapping Atchley vectors to codewords...'

seqs = []
for line in open(filename,"r"):
    line = line.rstrip("\n")
    seqs.append(line)

while counter < howmany:

    ## Sample q times to generate freq dist over codewords
    while count < q:
        pickseq = random.randint(0,len(seqs)-1)
        m = len(seqs[pickseq])
        if m > p:
            # start of p-mer must be located p steps from end
            picktriplet = random.randint(0,m-p)

            x = seqs[pickseq][picktriplet:picktriplet+p]
            af = str(atchley_factor(x))[1:-1]
            res = af.split(", ")
            vector = [eval(x) for x in res]
            v = np.array(vector)
            dist = (codewords - v)**2
            dist = np.sum(dist,axis=1)
            dist = np.sqrt(dist)
            ind = np.where(dist == dist.min())[0][0]
            histocount[ind]+=1
            count += 1

    print >> outfile, str(histocount)[1:-1]
    print counter
    histocount = [0]*len(codewords)
    count = 0
    counter += 1


## End sampling

outfile.close()
timed = time.time() - t0
print 'Finished in: ',timed,'seconds'



## SCRIPT 2

path = "/home/path/to/files/"

unt_1 <- read.table(paste(path,"results_unt_1.txt", sep=""), header=FALSE, sep=",")
unt_2 <- read.table(paste(path,"results_unt_2.txt", sep=""), header=FALSE, sep=",")
unt_3 <- read.table(paste(path,"results_unt_3.txt", sep=""), header=FALSE, sep=",")
unt_4 <- read.table(paste(path,"results_unt_4.txt", sep=""), header=FALSE, sep=",")
unt_5 <- read.table(paste(path,"results_unt_5.txt", sep=""), header=FALSE, sep=",")
unt_6 <- read.table(paste(path,"results_unt_6.txt", sep=""), header=FALSE, sep=",")

d5_1 <- read.table(paste(path,"results_d5_1.txt", sep=""), header=FALSE, sep=",")
d5_2 <- read.table(paste(path,"results_d5_2.txt", sep=""), header=FALSE, sep=",")
d5_3 <- read.table(paste(path,"results_d5_3.txt", sep=""), header=FALSE, sep=",")
d5_4 <- read.table(paste(path,"results_d5_4.txt", sep=""), header=FALSE, sep=",")
d5_5 <- read.table(paste(path,"results_d5_5.txt", sep=""), header=FALSE, sep=",")
d5_6 <- read.table(paste(path,"results_d5_6.txt", sep=""), header=FALSE, sep=",")

d14_1 <- read.table(paste(path,"results_d14_1.txt", sep=""), header=FALSE, sep=",")
d14_2 <- read.table(paste(path,"results_d14_2.txt", sep=""), header=FALSE, sep=",")
d14_3 <- read.table(paste(path,"results_d14_3.txt", sep=""), header=FALSE, sep=",")
d14_4 <- read.table(paste(path,"results_d14_4.txt", sep=""), header=FALSE, sep=",")
d14_5 <- read.table(paste(path,"results_d14_5.txt", sep=""), header=FALSE, sep=",")
d14_6 <- read.table(paste(path,"results_d14_6.txt", sep=""), header=FALSE, sep=",")

m2_1 <- read.table(paste(path,"results_m2_1.txt", sep=""), header=FALSE, sep=",")
m2_2 <- read.table(paste(path,"results_m2_2.txt", sep=""), header=FALSE, sep=",")
m2_3 <- read.table(paste(path,"results_m2_3.txt", sep=""), header=FALSE, sep=",")
m2_4 <- read.table(paste(path,"results_m2_4.txt", sep=""), header=FALSE, sep=",")
m2_5 <- read.table(paste(path,"results_m2_5.txt", sep=""), header=FALSE, sep=",")
m2_6 <- read.table(paste(path,"results_m2_6.txt", sep=""), header=FALSE, sep=",")

library("e1071")
numpersample <- 6
kernelfn <- "linear"

####################
## TEST ON UNT 1-6 ##
####################

for (i in 1:numpersample){

v <- c(1:numpersample)
v <- v[-i]

## BUILD TEST SET ##

test <- eval(as.name(paste("unt_",i,sep="")))
test <- as.data.frame(test)


## BUILD TRAINING SET ##

training <- c()
for (j in 1:numpersample){
training <- rbind(training,eval(as.name(paste("d5_",j,sep=""))))
training <- as.data.frame(training)
}
numd5 <- dim(training)[1]

for (j in 1:numpersample){
training <- rbind(training,eval(as.name(paste("d14_",j,sep=""))))
training <- as.data.frame(training)
}
numd14 <- dim(training)[1]-numd5

for (j in 1:numpersample){
training <- rbind(training,eval(as.name(paste("m2_",j,sep=""))))
training <- as.data.frame(training)
```

```
}
numm2 <- dim(training)[1]-numd5-numd14

for (j in v){
training <- rbind(training,eval(as.name(paste("unt_",j,sep=""))))
training <- as.data.frame(training)
}
numunt <- dim(training)[1]-numd5-numd14-numm2

training <- cbind( c(rep(1,numd5),rep(2,numd14),rep(3,numm2),rep(0,numunt)), training)
colnames(training)[1] <- "class"


## CREATE SVM CLASSIFIER ##

cdr3model <- svm( class ~ ., data = training, type = "C-classification", kernel = kernelfn)


## TEST SVM WITH PREDICT() ##

predictions <- predict(cdr3model, test )
print(table(predictions))

}

######################
## TEST ON DAY5 1-3 ##
######################

for (i in 1:numpersample){

v <- c(1:numpersample)
v <- v[-i]

## BUILD TEST SET ##

test <- eval(as.name(paste("d5_",i,sep="")))
test <- as.data.frame(test)


## BUILD TRAINING SET ##

training <- c()
for (j in 1:numpersample){
training <- rbind(training,eval(as.name(paste("unt_",j,sep=""))))
training <- as.data.frame(training)
}
numunt <- dim(training)[1]

for (j in 1:numpersample){
training <- rbind(training,eval(as.name(paste("d14_",j,sep=""))))
training <- as.data.frame(training)
}
numd14 <- dim(training)[1]-numunt

for (j in 1:numpersample){
training <- rbind(training,eval(as.name(paste("m2_",j,sep=""))))
training <- as.data.frame(training)
}
numm2 <- dim(training)[1]-numunt-numd14

for (j in v){
training <- rbind(training,eval(as.name(paste("d5_",j,sep=""))))
training <- as.data.frame(training)
}
numd5 <- dim(training)[1]-numunt-numd14-numm2

training <- cbind( c(rep(0,numunt),rep(2,numd14),rep(3,numm2),rep(1,numd5)), training)
colnames(training)[1] <- "class"


## CREATE SVM CLASSIFIER ##

cdr3model <- svm( class ~ ., data = training, type = "C-classification", kernel = kernelfn)


## TEST SVM WITH PREDICT() ##

predictions <- predict(cdr3model, test )
print(table(predictions))

}

#######################
## TEST ON DAY14 1-3 ##
#######################

for (i in 1:numpersample){

v <- c(1:numpersample)
v <- v[-i]

## BUILD TEST SET ##

test <- eval(as.name(paste("d14_",i,sep="")))
test <- as.data.frame(test)


## BUILD TRAINING SET ##

training <- c()
for (j in 1:numpersample){
training <- rbind(training,eval(as.name(paste("unt_",j,sep=""))))
training <- as.data.frame(training)
}
numunt <- dim(training)[1]

for (j in 1:numpersample){
training <- rbind(training,eval(as.name(paste("d5_",j,sep=""))))
training <- as.data.frame(training)
}
numd5 <- dim(training)[1]-numunt
```

```
for (j in 1:numpersample){
training <- rbind(training,eval(as.name(paste("m2_",j,sep=""))))
training <- as.data.frame(training)
}
numm2 <- dim(training)[1]-numunt-numd5

for (j in v){
training <- rbind(training,eval(as.name(paste("d14_",j,sep=""))))
training <- as.data.frame(training)
}
numd14 <- dim(training)[1]-numunt-numd5-numm2

training <- cbind( c(rep(0,numunt),rep(1,numd5),rep(3,numm2),rep(2,numd14)), training)
colnames(training)[1] <- "class"


## CREATE SVM CLASSIFIER ##

cdr3model <- svm( class ~ ., data = training, type = "C-classification", kernel = kernelfn)


## TEST SVM WITH PREDICT() ##

predictions <- predict(cdr3model, test )
print(table(predictions))

}

#######################
## TEST ON MONTH2 1-3 ##
#######################

for (i in 1:numpersample){

v <- c(1:numpersample)
v <- v[-i]

## BUILD TEST SET ##

test <- eval(as.name(paste("m2_",i,sep="")))
test <- as.data.frame(test)


## BUILD TRAINING SET ##

training <- c()
for (j in 1:3){
training <- rbind(training,eval(as.name(paste("unt_",j,sep=""))))
training <- as.data.frame(training)
}
numunt <- dim(training)[1]

for (j in 1:numpersample){
training <- rbind(training,eval(as.name(paste("d5_",j,sep=""))))
training <- as.data.frame(training)
}
numd5 <- dim(training)[1]-numunt

for (j in 1:numpersample){
training <- rbind(training,eval(as.name(paste("d14_",j,sep=""))))
training <- as.data.frame(training)
}
numd14 <- dim(training)[1]-numunt-numd5

for (j in v){
training <- rbind(training,eval(as.name(paste("m2_",j,sep=""))))
training <- as.data.frame(training)
}
numm2 <- dim(training)[1]-numunt-numd5-numd14

training <- cbind( c(rep(0,numunt),rep(1,numd5),rep(2,numd14),rep(3,numm2)), training)
colnames(training)[1] <- "class"


## CREATE SVM CLASSIFIER ##

cdr3model <- svm( class ~ ., data = training, type = "C-classification", kernel = kernelfn)


## TEST SVM WITH PREDICT() ##

predictions <- predict(cdr3model, test )
print(table(predictions))

}
```

Supplementary Figures

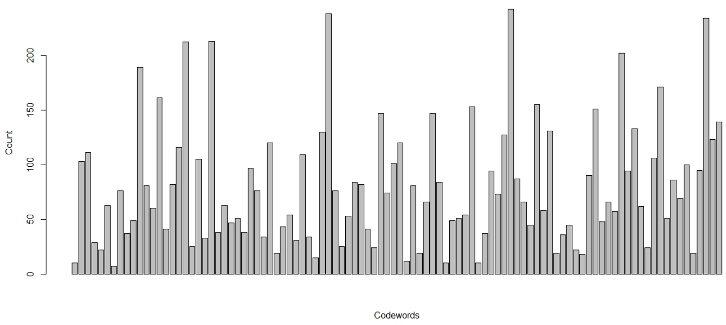

Figure 1: Frequency distribution of the $20^3 = 8000$ different possible amino acid triplets across the codebook. The number of triplets within each of the 100 codewords is shown.

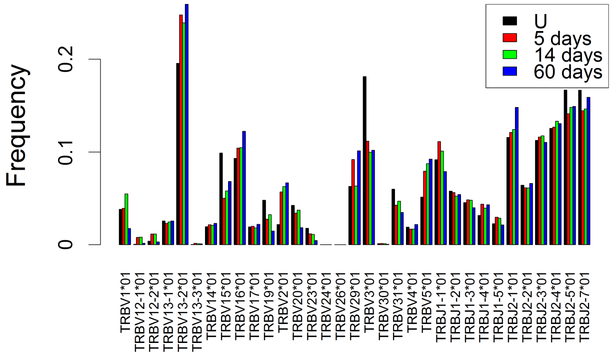

Figure 2: The frequency distribution of mouse V and J region usage within the different groups of mice. The frequencies for all mice in each experimental group were averaged.

Supplementary Tables

Table 2: Feasible triplets for each of the 100 codewords.

Codeword	Triplets
1	DSK, DSR, PSK, PSR, QSK, QSR, SDK, SDR, SSK, SSR
2	FEF, FEG, FEI, FEK, FEM, FEN, FER, FET, FEY, GEF, GEG, GEI, GEK, GEM, GEN, GER, GET, GEW, GEY, GFR, GMK, GMR, IEF, IEG, IEI, IEK, IEM, IEN, IER, IET, IEY, KEF, KEG, KEI, KEK, KEM, KEN, KER, KET, KEW, KEY, KFR, KMN, KMR, MEG, MEI, MEK, MEN, MER, MET, MEY, NEF, NEG, NEI, NEK, NEM, NEN, NER, NET, NEY, NFR, NMK, NMN, NMR, REF, REG, REI, REK, REM, REN, RER, RET, REW, REY, RFR, RMR, TEF, TEG, TEI, TEK, TEM, TEN, TER, TET, TEY, TFR, TMR, WEG, WEI, WEK, WEM, WEN, WER, WET, WEY, YEG, YEI, YEK, YEN, YER, YET, YEY, YMR
3	AFG, AFK, AFN, AFR, AFT, AFY, AIK, AIN, AIR, AIT, AIY, AKF, AKI, AKM, AKT, AMK, AMN, AMR, AMT, AMY, ANF, ANG, ANI, ANK, ANN, ANR, ANT, ANY, ATK, ATN, ATR, ATT, ATY, AYG, AYK, AYN, AYR, AYT, AYY, CFT, CFY, CNK, CNR, CNT, CNY, LFK, LFN, LFR, LFT, LFY, LIK, LIN, LIR, LIT, LIY, LKF, LKI, LMK, LMN, LMR, LMT, LNG, LNI, LNK, LNN, LNR, LNT, LNY, LTK, LTN, LTR, LTT, LTY, LYK, LYN, LYR, LYT, VFG, VFK, VFN, VFR, VFT, VFY, VIK, VIN, VIR, VIT, VIY, VKF, VKI, VMK, VMN, VMR, VMT, VNG, VNI, VNK, VNN, VNR, VNT, VNY, VTK, VTN, VTR, VTT, VTY, VYG, VYK, VYN, VYR, VYT
4	AGF, AGG, AGI, AGK, AGN, AGR, AGT, AGY, AIG, ATG, LGG, LGI, LGK, LGN, LGR, LGT, LGY, LIG, LTG, LYG, VGG, VGI, VGK, VGN, VGR, VGT, VGY, VIG, VTG
5	DFR, DFT, DIT, DMN, DMR, DMT, DTI, DYG, DYI, DYK, DYN, DYR, DYT, DYY, QYG, QYK, QYN, QYR, QYT, SYI, SYT, SYY
6	DAA, DAL, DAV, DCA, DCL, DCV, DHA, DHL, DHV, DLA, DLL, DLV, DPA, DVA, DVL, DVV, PAL, PHA, PHL, PLL, PVL, QAA, QAL, QCA, QCL, QCV, QHA, QLA, QLL, QLV, QPA, QVA, QVL, QVV, SAA, SAC, SAH, SAL, SAV, SCA, SCC, SCL, SCV, SEA, SEL, SEV, SHA, SHC, SHL, SHV, SLA, SLC, SLH, SLL, SLV, SPA, SPL, SPV, SVA, SVC, SVH, SVL, SVV
7	AEG, CEG, LEG, LFG, LMG, VEG, VMG
8	DAH, DAQ, DCC, DCH, DCQ, DEA, DEC, DED, DEH, DEL, DEP, DEQ, DES, DEV, DFH, DFQ, DHC, DHH, DHQ, DIH, DKC, DKH, DKP, DLH, DMH, DMQ, DNH, DVH, DVQ, DWC, DWH, DWL, DWQ, HWH, PEC, PEH, PHH, QAH, QAQ, QCH, QCQ, QEC, QED, QEH, QEL, QEP, QEQ, QES, QEV, QFH, QFQ, QHC, QHH, QHQ, QIH, QKC, QKH, QMH, QMQ, QNH, QVH, QVQ, QWH, QWQ, SCH, SEC, SEH, SEP, SEQ, SFH, SHH, SIH, SKH, SMH, SWC, SWH

Continued on next page

Table 2 – continued from previous page

Codeword	Triplets
9	DGD, DGH, DGP, DGQ, DID, DIQ, DND, DNP, DNQ, DTD, DTH, DTP, DTQ, DYD, DYH, DYP, DYQ, QGQ, QID, QIQ, QNQ, QTD, QTH, QTP, QTQ, QYD, QYP, QYQ, SGD, SGP, SGQ, SND, SNQ, STD, STQ, SYD, SYQ
10	GFG, GGG, GIG, GMG, GTG, GYG, IGG, IIG, IIT, ING, ITG, IYG, KFG, KGG, KIG, KIT, KNG, KTG, KYG, NFG, NGG, NIG, NIT, NNG, NTG, NTT, NYG, RFG, RGG, RIG, RIT, RMG, RNG, RTG, RTT, RYG, TFG, TGG, TIG, TIT, TNG, TTG, TTT, TYG, YGG, YIG, YNG, YTG, YYG
11	FDF, FDG, FDI, FDK, FDM, FDN, FDR, FDT, FDW, FDY, FQG, FQK, FQN, FQR, FQT, FQY, FSY, GDF, GDG, GDI, GDK, GDM, GDN, GDR, GDT, GDY, GHM, GQF, GQG, GQI, GQK, GQM, GQN, GQR, GQT, GQW, GQY, IDF, IDG, IDI, IDK, IDM, IDN, IDR, IDT, IDW, IDY, IQE, IQF, IQG, IQI, IQK, IQM, IQN, IQR, IQT, IQW, IQY, ISY, KDE, KDF, KDG, KDI, KDK, KDM, KDN, KDR, KDT, KDW, KDY, KQG, KQK, KQN, KQR, KQT, KQY, KSR, KSY, MDE, MDG, MDI, MDK, MDN, MDR, MDT, MDY, MQG, MQK, MQN, MQR, MQT, MQY, MSR, MSY, NDF, NDG, NDI, NDK, NDM, NDN, NDR, NDT, NDW, NDY, NQE, NQF, NQG, NQI, NQK, NQM, NQN, NQR, NQT, NQW, NQY, NSY, RDE, RDF, RDG, RDI, RDK, RDM, RDN, RDR, RDT, RDW, RDY, RQE, RQF, RQG, RQI, RQK, RQM, RQN, RQR, RQT, RQW, RQY, RSR, RSY, TDF, TDG, TDI, TDK, TDM, TDN, TDR, TDT, TDW, TDY, TQE, TQF, TQG, TQI, TQK, TQM, TQN, TQR, TQT, TQW, TQY, TSY, WDN, WDR, WDT, WDY, WQG, WQR, YDF, YDG, YDI, YDK, YDM, YDN, YDR, YDT, YDW, YDY, YQF, YQG, YQI, YQK, YQM, YQN, YQR, YQT, YQW, YQY, YSY
12	AFE, AGE, AIE, AKE, AME, ANE, ARE, ATE, AYE, CFE, CGE, CGK, CGM, CIE, CIK, CKE, CME, CNE, CRE, CTE, CTK, CWE, CYE, CYK, EFE, EGE, EIE, EKE, EME, ENE, ERE, ETE, EWE, EYE, FGE, FNE, FRE, FTE, HCE, HFE, HGE, HGK, HGW, HIE, HKE, HNE, HRE, HTE, HTK, HYE, IGE, LFE, LGE, LIE, LKE, LNE, LRE, LTE, LYE, MGE, MRE, VCE, VFE, VGE, VIE, VKE, VME, VNE, VRE, VTE, VYE, WFE, WGE, WIE, WKE, WME, WNE, WRE, WTE, WWE, WYE
13	AKA, AKC, AKH, AKL, AKV, ANC, ANH, ARA, ARC, ARH, ARL, ARV, ATC, CKA, CKC, CKH, CKL, CKV, CRA, CRC, CRH, CRL, CRP, CRV, HKA, HKC, HKH, HKL, HKV, HRA, HRC, HRH, HRL, HRP, HRV, LKA, LKC, LKH, LKL, LKV, LNC, LNH, LRA, LRC, LRH, LRL, LRV, VKA, VKC, VKH, VKL, VKV, VNC, VNH, VRA, VRC, VRH, VRL, VRV, WRH
14	ACE, ACF, ACG, ACI, ACK, ACM, ACN, ACR, ACT, ACW, ACY, AHC, AHE, AHF, AHG, AHI, AHK, AHM, AHN, AHR, AHT, AHW, AHY, AMG, AWC, AWE, AWF, AWG, AWI, AWK, AWM, AWN, AWR, AWT, AWY, CCG, CCI, CCK, CCN, CCR, CCT, CCY, CFK, CFN, CFR, CHG, CHI, CHK, CHN, CHR, CHT, CHY, CMG, CMK, CMN, CMR, CWF, CWG, CWI, CWK, CWM, CWN, CWR, CWT, CWY, ECR, EWF, EWI, EWK, EWN, EWR, EWT, EWY, HCG, HCK, HCN, HCR, HCT, HFR, HHF, HHG, HHI, HHK, HHN, HHR, HHT, HHY, HMG, HMR, HWG, HWK, HWN, HWR, HWT, HWY, LCE, LCF, LCG, LCI, LCK, LCM, LCN, LCR, LCT, LCY, LHC, LHE, LHF, LHG, LHI, LHK, LHM, LHN, LHR, LHT, LHY, LWC, LWE, LWF, LWG, LWI, LWK, LWN, LWR, LWT, LWY, PCR, PHR, PWK, PWN, PWR, PWT, PWY, VCF, VCG, VCI, VCK, VCM, VCN, VCR, VCT, VCY, VHE, VHF, VHG, VHI, VHK, VHM, VHN, VHR, VHT, VHY, VWC, VWE, VWG, VWI, VWK, VWN, VWR, VWT, VWY
15	DCG, DCI, DCK, DCN, DCR, DCT, DFG, DHG, DHT, DMG, DWG, DWI, DWK, DWN, DWR, DWT, DWY, PCG, PHG, PWG, QCG, QCR, QCT, QFG, QHG, QHT, QMG, QWG, QWK, QWN, QWR, QWT, QWY, SCG, SWG, SWI, SWK, SWN, SWR, SWT, SWY
16	FKG, FKI, FKN, FKR, FKT, FKY, FRG, FRI, FRN, FRR, FRT, FRY, GKG, GRG, IKG, IKN, IKR, IKT, IRG, IRI, IRR, IRT, IRY, KKG, KKI, KKN, KKR, KKT, KRF, KRG, KRI, KRN, KRR, KRT, KRY, MKG, MKK, MKN, MKR, MKT, MKY, MRG, MRN, MRR, MRT, MRY, NKG, NKR, NKT, NRG, NRI, NRR, NRT, NRY, RKG, RKN, RKR, RKT, RRG, RRI, RRN, RRR, RRT, RRY, TKG, TKR, TKT, TRG, TRI, TRR, TRT, TRY, YKG, YKN, YKR, YKT, YKY, YRG, YRN, YRR, YRT, YRY
17	FDE, FSE, FSF, FSI, FSK, FSM, FSN, FSR, FST, FSW, GDE, GDW, GSA, GSC, GSE, GSF, GSG, GSH, GSI, GSK, GSM, GSN, GSR, GST, GSV, GSW, GSY, IDE, ISC, ISE, ISF, ISG, ISH, ISI, ISK, ISM, ISN, ISR, IST, ISW, KSC, KSE, KSF, KSG, KSH, KSI, KSK, KSM, KSN, KST, KSW, MSE, MSF, MSG, MSI, MSK, MSM, MSN, MST, MSW, NDE, NSC, NSE, NSF, NSG, NSH, NSI, NSK, NSM, NSN, NSR, NST, NSW, RSA, RSC, RSE, RSF, RSG, RSH, RSI, RSK, RSM, RSN, RST, RSV, RSW, TDE, TSA, TSC, TSE, TSF, TSG, TSH, TSI, TSK, TSM, TSN, TSR, TST, TSV, TSW, YDE, YSA, YSC, YSE, YSF, YSG, YSH, YSI, YSK, YSM, YSN, YSR, YST, YSV, YSW
18	CFH, CMH, CWH, CWQ, ECH, EVH, FAH, FCH, FCL, FCP, FCQ, FFH, FFQ, FHD, FHH, FHP, FHQ, FIH, FIQ, FLH, FMH, FMP, FMQ, FPH, FVH, FWH, FWP, FWQ, GAC, GAH, GCC, GCH, GCL, GCP, GCQ, GEH, GFC, GFH, GFP, GFQ, GHC, GHH, GHP, GHQ, GIC, GIH, GIQ, GKH, GLH, GMC, GMD, GMH, GMP, GMQ, GNH, GTH, GVC, GVH, GWC, GWD, GWH, GWP, GWQ, GYH, IAH, ICH, ICL, ICP, ICQ, IFH, IFQ, IHC, IHH, IHP, IHQ, IIH, IIQ, ILH, IMH, IMP, IMQ, IPH, IVC, IVH, IWD, IWH, IWP, IWQ, KAH, KCH, KCQ, KFH, KHH, KHQ, KIH, KKH, KMH, KVH, KWC, KWH, KWQ, MAH, MCH, MCL, MCQ, MFH, MFQ, MHH, MHP, MHQ, MIH, MLH, MMH, MVH, MWH, MWP, MWQ, NAH, NCC, NCH, NCQ, NFC, NFH, NFQ, NHC, NHH, NHP, NHQ, NIH, NIQ, NKH, NLH, NMC, NMH, NMQ, NVH, NWC, NWD, NWH, NWP, NWQ, RAH, RCH, RCL, RFC, RFH, RHH, RHQ, RIH, RKH, RMC, RMH, RVH, RWC, RWH, RWQ, TAC, TAH, TCC, TCH, TCL, TCP, TCQ, TFC, TFH, TFQ, THC, THH, THP, THQ, TIH, TIQ, TKH, TLH, TMC, TMH, TMP, TMQ, TVC, TVH, TWC, TWD, TWH, TWP, TWQ, WCH, WCQ, WFH, WHQ, WIH, WMH, WWH, WWP, WWQ, YAH, YCC, YCH, YCL, YCP, YCQ, YFH, YHC, YHH, YHP, YIH, YIQ, YMH, YVH, YWC, YWH, YWP, YWQ
19	SFG, SFR, SFT, SFY, SGG, SGR, SGT, SIG, SII, SIR, SIT, SIY, SMG, SMR, SMT, SMY, SNG, SNR, SNT, STG, STI, STR, STT, SYG, SYR
20	AAA, AAL, AAV, ACA, ACC, ACH, ACL, ACV, AGA, AGC, AGL, AGV, AHA, AHL, AHV, ALA, ALL, ALV, ANL, ANV, APA, APC, APE, APH, APL, APV, AVA, AVL, AVV, CCL, CCV, CHA, CHL, CHV, CVL, EPA, EPL, EPV, FCA, FCV, HCA, HCL, HCV, HHA, HHL, HHV, HVA, HVL, ICA, LAA, LAL, LAV, LCA, LCC, LCH, LCL, LCV, LGA, LGC, LGL, LGV, LHA, LHL, LHV, LLA, LLL, LLV, LNL, LNV, LPA, LPC, LPE, LPL, LPV, LVA, LVL, LVV, PPL, VAA, VAL, VAV, VCA, VCC, VCH, VCL, VCV, VGA, VGC, VGL, VGV, VHA, VHL, VHV, VLA, VLL, VLV, VNV, VPA, VPC, VPE, VPL, VPV, VVA, VVL, VVV
21	DEE, DFE, DFK, DGE, DIE, DIK, DME, DMK, DNE, DNK, DTE, DWE, DYE, PEE, PFE, PGE, PIE, PKE, PME, PNE, PTE, PWE, PYE, QCE, QEE, QFE, QGE, QIE, QME, QNE, QTE, QWE, QYE
22	AAC, AAH, AAW, ALC, ALH, ALW, AVC, AVH, AVW, CAA, CAC, CAE, CAF, CAH, CAI, CAK, CAL, CAM, CAN, CAV, CAW, CAY, CCC, CCE, CCF, CCH, CCM, CCW, CEC, CEW, CFC, CFW, CHC, CHE, CHF, CHH, CHM, CHW, CIC, CIH, CIW, CKW, CLA, CLC, CLE, CLF, CLH, CLK, CLL, CLM, CLN, CLV, CLW, CLY, CMW, CPF, CPM, CPW, CQW, CVC, CVE, CVF, CVH, CVK, CVM, CVN, CVV, CVW, CVY, CWC, CWW, EAC, EAH, EAW, ELH, FAC, FAW, FLC, FLW, FVC, FVW, HAA, HAC, HAE, HAF, HAH, HAI, HAK, HAL, HAM, HAN, HAV, HAW, HCC, HCH, HCW, HEC, HEW, HHC, HHE, HHH, HHM, HHW, HIH, HKW, HLA, HLC, HLE, HLF, HLH, HLK, HLL, HLM, HLV, HLW, HPW, HVC, HVE, HVF, HVH, HVK, HVM, HVV, HVW, IAC, IAW, ILC, ILW, IVW, LAC, LAE, LAH, LAW, LCW, LHW, LLC, LLE, LLH, LLW, LPW, LVC, LVE, LVH, LVW, MAC, MAW, MLC, MLW, MVC, MVW, NAW, PAH, PEW, VAC, VAE, VAF, VAH, VAM, VAW, VCW, VHC, VHW, VLC, VLE, VLH, VLW, VPW, VVC, VVE, VVH, VVW, WAC, WAF, WAH, WAI, WAK, WAM, WAN, WAT, WAV, WAW, WAY, WCC, WCF, WCM, WCW, WEC, WEF, WEW, WHC, WHH, WHW, WLA, WLC, WLF, WLH, WLI, WLK, WLM, WLN, WLT, WLV, WLW, WPW, WVC, WVF, WVH, WVI, WVK, WVM, WVN, WVV, WVW
23	AGP, AGS, AIP, AIS, AKS, ANP, ANS, ARS, ATP, ATS, AYP, AYS, LFP, LGP, LGS, LIP, LIS, LKS, LNP, LNS, LRS, LTP, LTS, LYP, LYS, VFP, VGP, VGS, VIP, VIS, VKS, VNP, VNS, VRS, VTP, VTS, VYP, VYS
24	FGD, FGH, FGQ, FID, FND, FNQ, FRD, FRQ, FTD, FTQ, FYD, FYQ, GGH, IGD, IGH, IGQ, IID, IKQ, IND, INQ, IRD, IRQ, ITD, ITQ, IYD, IYQ, MGD, MGH, MGQ, MID, MIQ, MND, MNQ, MRQ, MTD, MTQ, MYD, MYQ, NGH, NGQ, NNQ, NTQ, NYQ, TGD, TGH, TGQ, TNQ, TRQ, TTD, TTH, TTQ, TYD, TYQ, YGD, YGH, YGQ, YND, YNQ, YRQ, YTD, YTH, YTQ, YYQ
25	DGW, DNW, DTW, SCW, SFC, SFK, SFN, SFW, SGC, SGF, SGH, SGI, SGK, SGM, SGN, SGW, SGY, SIC, SIK, SIN, SIW, SKW, SMK, SMN, SMW, SNC, SNF, SNH, SNI, SNK, SNM, SNN, SNW, SNY, SRW, STC, STF, STH, STK, STM, STN, STW, STY, SWW, SYH, SYN, SYW

Continued on next page

Table 2 – continued from previous page

Codeword	Triplets
26	AEE, AEF, AEI, AEK, AEM, AEN, AER, AET, AEW, AEY, CEE, CEF, CEI, CEK, CEM, CEN, CER, CET, CEY, EEE, EEF, EEG, EEI, EEK, EEM, EEN, EER, EET, EEY, HEE, HEI, LEE, LEF, LEI, LEK, LEM, LEN, LER, LET, LEW, LEY, VEE, VEF, VEI, VEK, VEM, VEN, VER, VET, VEW, VEY
27	EKA, EKC, EKH, EKL, EKP, EKV, ERA, ERC, ERH, ERL, ERP, ERV, FKA, FKL, FKV, FRA, FRL, FRV, IRA, IRL, IRV, KKA, KKC, KKL, KKV, KRA, KRC, KRH, KRL, KRV, MKA, MKL, MKV, MRA, MRL, MRV, WKL, WKV
28	AAF, AAG, AAI, AAK, AAM, AAN, AAR, AAT, AAY, ALF, ALG, ALI, ALK, ALM, ALN, ALR, ALT, ALY, AVF, AVG, AVI, AVK, AVM, AVN, AVR, AVT, AVY, CAG, CAR, CAT, CLG, CLR, CLT, CVG, CVR, CVT, EAG, EAR, EVG, HAG, HAR, HAT, HLG, HLR, HVG, HVR, LAF, LAG, LAI, LAK, LAM, LAN, LAR, LAT, LAY, LLF, LLG, LLI, LLK, LLM, LLN, LLR, LLT, LLY, LVF, LVG, LVI, LVK, LVM, LVN, LVR, LVT, LVY, VAG, VAI, VAK, VAN, VAR, VAT, VAY, VLG, VLI, VLK, VLN, VLR, VLT, VLY, VVG, VVI, VVK, VVN, VVR, VVT, VVY, WAG, WLG, WVG
29	EES, FES, FFD, FFS, FIS, FKD, FKS, FMD, FMS, FNS, FRS, FTS, FYS, GFS, GKS, GMS, GWS, IES, IFD, IFS, IIS, IKD, IKS, IMD, IMS, INS, IRS, ITS, IYS, MCS, MES, MFD, MFS, MHS, MIS, MKS, MMS, MNS, MRS, MTS, MWS, MYS, NFS, NIS, NKS, NMS, NNS, NRS, NTS, NWS, NYS, TES, TFD, TFS, THS, TID, TIS, TKD, TKS, TMS, TND, TNS, TRS, TTS, TWS, TYS, YFS, YHS, YIS, YKS, YMS, YNS, YRS, YTS, YWS, YYS
30	DGF, DGG, DGI, DGK, DGM, DGN, DGR, DGT, DGY, DIG, DIN, DIR, DTG, DTK, DTN, DTR, DTT, DTY, QGF, QGG, QGI, QGK, QGN, QGR, QGT, QGY, QIG, QIR, QTG, QTK, QTN, QTR, QTT, QTY
31	CPG, CPI, CPK, CPN, CPR, CPT, CPY, FPC, FPE, FPF, FPG, FPI, FPK, FPM, FPN, FPR, FPT, FPY, GPC, GPF, GPG, GPI, GPK, GPM, GPN, GPR, GPT, GPW, GPY, IPC, IPE, IPF, IPG, IPI, IPK, IPM, IPN, IPR, IPT, IPW, IPY, KPC, KPE, KPF, KPG, KPH, KPI, KPK, KPN, KPR, KPT, KPW, KPY, MPC, MPF, MPG, MPI, MPK, MPN, MPR, MPT, MPY, NPC, NPE, NPF, NPG, NPH, NPI, NPK, NPM, NPN, NPR, NPT, NPW, NPY, RPC, RPE, RPF, RPG, RPH, RPI, RPK, RPM, RPN, RPR, RPT, RPW, RPY, TPC, TPE, TPF, TPG, TPH, TPI, TPK, TPM, TPN, TPR, TPT, TPW, TPY, WPG, WPI, WPK, WPN, WPR, WPT, WPY, YPC, YPF, YPG, YPH, YPI, YPK, YPM, YPN, YPR, YPT, YPW, YPY
32	ADS, APS, ASD, ASP, ASS, CSS, EDS, ESD, ESS, FSS, HSS, LDS, LPS, LSS, PSS, QSS, VPS, VSS, WSS
33	DKI, DNF, DNG, DNI, DNN, DNR, DNT, DNY, HKF, HKI, HKM, HKT, HKY, HNG, HNI, HNK, HNN, HNR, HNT, HNY, HYR, PKM, QFR, QKF, QKG, QKI, QKK, QKM, QKN, QKR, QKT, QKY, QNF, QNG, QNI, QNK, QNN, QNR, QNT, QNY, QRI, QRT, QRY
34	CGD, CGQ, CGS, CNS, CRS, CTS, CYS, FGS, HGD, HGP, HGQ, HGS, HIS, HTP, HTS, MGS, WCD, WFD, WFP, WFQ, WFS, WGD, WGP, WGQ, WGS, WID, WIP, WIQ, WIS, WKD, WKP, WKQ, WKS, WMD, WMP, WMQ, WMS, WND, WNP, WNQ, WNS, WRD, WRP, WRQ, WRS, WTD, WTP, WTQ, WTS, WWD, WYD, WYP, WYQ, WYS
35	DKG, DKN, DKR, DKT, DKY, DRG, DRN, DRR, DRT, DRY, QRG, QRN, QRR, SKF, SKG, SKI, SKK, SKM, SKN, SKR, SKT, SKY, SRF, SRG, SRI, SRK, SRM, SRN, SRR, SRT, SRY
36	CFG, CGG, CGN, CGR, CGT, CGY, CIG, CIN, CIR, CIT, CIY, CNG, CNN, CTG, CTN, CTR, CTT, CTY, CYG, CYN, CYR, CYT, HFG, HGG, HGI, HGN, HGR, HGT, HGY, HIG, HIR, HTG, HTN, HTR, HTT, HTY, HYG, PFG, PFK, PFN, PFR, PFT, PFY, PGF, PGG, PGI, PGK, PGM, PGN, PGR, PGT, PGY, PIG, PIK, PIN, PIR, PIT, PIY, PKF, PKG, PKI, PKK, PKN, PKR, PKT, PKW, PKY, PMG, PMK, PMN, PMR, PMT, PMY, PNF, PNG, PNI, PNK, PNM, PNN, PNR, PNT, PNW, PNY, PRF, PRG, PRI, PRK, PRM, PRN, PRR, PRT, PRW, PRY, PTF, PTG, PTI, PTK, PTM, PTN, PTR, PTT, PTW, PTY, PYG, PYK, PYN, PYR, PYT, PYY
37	DDE, DDW, DHE, DHW, DPE, DPF, DPM, DPW, DQE, DQW, DSE, LQE, PDE, PPE, PQE, PSE, QHE, QPE, QPW, QQE, QSE, SDE, SDW, SHE, SHW, SPE, SPH, SPM, SPW, SQE, SQF, SQM, SQW, SSE
38	GFD, GGD, GGQ, GID, GKD, GKQ, GND, GNQ, GRD, GRQ, GTD, GTQ, GYD, GYQ, TRD
39	AFC, AFF, AFI, AFM, AFW, AGM, AIC, AIF, AII, AIM, AIW, AMC, AMF, AMI, AMM, AMW, ANM, ANW, ATF, ATI, ATM, ATW, AWW, AYF, AYI, AYM, AYW, CFF, CFI, CFM, CIF, CII, CIM, CKM, CMC, CMF, CMI, CMM, CMT, CMY, CNF, CNI, CNM, CTF, CTI, CTM, CYF, CYI, CYM, CYW, CYY, EMM, LFC, LFF, LFI, LFM, LFW, LGF, LGM, LGW, LIC, LIF, LII, LIM, LIW, LKM, LKW, LMC, LME, LMF, LMI, LMM, LMW, LMY, LNF, LNM, LNW, LRM, LTC, LTF, LTI, LTM, LTW, LWM, LWW, LYC, LYF, LYI, LYM, LYW, LYY, VFC, VFF, VFI, VFM, VFW, VGF, VGM, VGW, VIC, VIF, VII, VIM, VIW, VKM, VKW, VMC, VMF, VMI, VMM, VMW, VMY, VNF, VNM, VNW, VRM, VTC, VTF, VTI, VTM, VTW, VWF, VWM, VWW, VYC, VYF, VYI, VYM, VYW, VYY
40	ECG, EWG, FCF, FCG, FCI, FCK, FCM, FCN, FCR, FCT, FFG, FFR, FHG, FHI, FHK, FHN, FHR, FHT, FHY, FMG, FMN, FMR, FMT, FWF, FWG, FWI, FWK, FWM, FWN, FWR, FWT, FWY, GCF, GCG, GCI, GCN, GCR, GCT, GHF, GHG, GHI, GHK, GHN, GHR, GHT, GWF, GWG, GWI, GWK, GWN, GWR, GWT, GWY, ICF, ICG, ICI, ICK, ICN, ICR, ICT, IFG, IFN, IFR, IFT, IHF, IHG, IHI, IHK, IHN, IHR, IHT, IMG, IMN, IMR, IMT, IWF, IWG, IWI, IWK, IWM, IWN, IWR, IWT, IWY, KCG, KCR, KHG, KHN, KHR, KHT, KMG, KWF, KWG, KWI, KWK, KWM, KWN, KWR, KWT, KWY, MCA, MCF, MCG, MCI, MCK, MCN, MCR, MCT, MFG, MHG, MHK, MHN, MHR, MHT, MHY, MMG, MMR, MMT, MWF, MWG, MWI, MWK, MWM, MWN, MWR, MWT, MWY, NCG, NCI, NCK, NCN, NCR, NCT, NCY, NFN, NHF, NHG, NHI, NHK, NHN, NHR, NHT, NHY, NMG, NWF, NWG, NWI, NWK, NWM, NWN, NWR, NWT, NWY, RCG, RHG, RHR, RWF, RWG, RWI, RWK, RWN, RWR, RWT, RWY, TCF, TCG, TCI, TCK, TCN, TCR, TCT, THF, THG, THI, THK, THN, THR, THT, TMG, TWF, TWG, TWI, TWK, TWM, TWN, TWR, TWT, TWY, WCG, WCI, WCK, WCN, WCR, WCT, WCY, WHG, WHI, WHK, WHN, WHR, WHT, WHY, WWF, WWG, WWI, WWK, WWM, WWN, WWR, WWT, WWY, YCF, YCG, YCI, YCK, YCN, YCR, YCT, YFG, YFR, YHF, YHG, YHI, YHK, YHM, YHN, YHR, YHT, YHY, YMG, YWF, YWG, YWI, YWK, YWN, YWR, YWT, YWY
41	DDA, DDC, DDD, DDH, DDL, DDP, DDQ, DDS, DDV, DPC, DPH, DPL, DPP, DPV, DQA, DQC, DQH, DQL, DQP, DQQ, DQV, PDC, PDH, PDL, PDP, PDV, PPH, PPP, PQA, PQC, PQH, PQL, PQP, PQV, QDA, QDC, QDH, QDL, QDP, QDQ, QDS, QDV, QHL, QHV, QPC, QPH, QPL, QPV, QQA, QQC, QQH, QQL, QQP, QQQ, QQS, QQV, QSP, SDA, SDC, SDD, SDH, SDL, SDP, SDQ, SDS, SDV, SHP, SPC, SPP, SQA, SQC, SQH, SQL, SQP, SQQ, SQV
42	DSF, DSG, DSI, DSM, DSN, DST, DSW, DSY, PSF, PSM, PSW, PSY, SDF, SDI, SDM, SDT, SDY, SSF, SSG, SSI, SSM, SSN, SST, SSW, SSY
43	KFD, KFQ, KID, KIQ, KKD, KKQ, KMD, KMQ, KMS, KND, KNQ, KRD, KRQ, KTD, KWD, KYD, KYQ, MKD, MMD, MRD, NFD, NID, NKD, NMD, NND, NRD, NRQ, NTD, NYD, RFD, RFQ, RID, RIQ, RKD, RKQ, RMD, RMP, RMQ, RMS, RND, RNQ, RRD, RRH, RRQ, RTD, RTQ, RWD, RYD, RYQ, TMD, YID, YRD, YYD
44	GGF, GGI, GGK, GGM, GGN, GGR, GGT, GGY, GIK, GIN, GIR, GIY, GTK, GTN, GTR, GTY, IGK, IGN, IGR, IGT, IGY, IIN, IIR, ITK, ITN, ITR, ITY, KGK, KGM, KGN, KGR, KGY, KTK, NGK, NGN, NGR, NGT, NGY, NIN, NIR, NIY, NTK, NTN, NTR, NTY, RGF, RGI, RGK, RGM, RGN, RGR, RGT, RGY, RIK, RIN, RIR, RIY, RTK, RTN, RTR, RTY, TGF, TGI, TGK, TGM, TGN, TGR, TGT, TGY, TIK, TIN, TIR, TIY, TTK, TTN, TTR, TTY, YGK, YGN, YGR, YGT, YGY, YIR, YTR
45	AAS, ALS, AVS, CAD, CAP, CAQ, CAS, CCD, CCP, CCQ, CCS, CES, CFS, CHD, CHP, CHS, CIS, CLD, CLP, CLQ, CLS, CPS, CVD, CVP, CVQ, CVS, ELS, FAS, FLS, FVS, HAD, HAQ, HAS, HCD, HCQ, HCS, HES, HFS, HHS, HLD, HLQ, HLS, HPS, HVD, HVQ, HVS, LAD, LAS, LLS, LVD, LVS, MAS, PAD, PAS, PLS, PVS, QAS, VAD, VAS, VLD, VLS, VVD, VVS, WAD, WAP, WAQ, WAS, WCP, WCS, WES, WHD, WHP, WHS, WLD, WLP, WLQ, WLS, WPS, WVD, WVP, WVQ, WVS
46	DFD, DFP, DFS, DIP, DMD, DMP, DMS, DWD, DWP, DWS, PWP, QFD, QMD, QMP, QMS, QWD, QWS, SED, SES, SFD, SFP, SFQ, SFS, SID, SIP, SIQ, SIS, SMD, SMP, SMQ, SMS, SNP, SNS, STP, STS, SWD, SWP, SWQ, SWS, SYP, SYS
47	GED, GEP, GEQ, GES, IED, KED, KEH, KEP, KEQ, KES, NED, NEP, NEQ, NES, NKQ, RED, REH, REP, REQ, RES, TED, TEP, TEQ, TKQ

Continued on next page

Table 2 – continued from previous page

Codeword	Triplets
48	FFL, FFV, FGL, FGV, FIA, FIL, FIV, FNL, FNV, FTA, FTL, FTV, GFL, GFV, GGA, GGL, GGV, GIA, GIL, GIV, GKL, GNL, GNV, GRA, GRL, GRV, GTA, GTL, GTV, IFL, IGA, IGL, IGV, IIA, IIL, IIV, INL, INV, ITA, ITL, ITV, KAL, KCL, KFC, KFL, KFV, KGA, KGL, KGV, KIA, KIC, KIL, KIV, KNL, KNV, KTA, KTC, KTL, KTV, MGA, MGL, MGV, MNL, MNV, MTA, MTV, NAL, NCA, NCL, NCV, NFL, NFV, NGA, NGL, NGV, NIA, NIC, NIL, NIV, NKA, NKL, NKV, NNL, NNV, NRA, NRL, NRV, NTA, NTC, NTH, NTL, NTV, NVL, RFL, RFV, RGA, RGL, RGV, RIA, RIC, RIL, RIV, RKA, RKL, RNL, RNV, RRA, RRC, RRL, RRV, RTA, RTC, RTL, RTV, TFL, TGA, TGL, TGV, TIA, TIL, TIV, TNL, TNV, TRA, TRL, TRV, TTA, TTL, TTV, YCV, YFL, YFV, YGA, YGL, YGV, YIA, YIL, YIV, YKL, YNL, YNV, YRA, YRL, YRV, YTA, YTL, YTV
49	AKG, AKK, AKN, AKR, AKW, AKY, ARF, ARG, ARI, ARK, ARM, ARN, ARR, ART, ARW, ARY, CKF, CKG, CKI, CKK, CKN, CKR, CKT, CKY, CRF, CRG, CRI, CRK, CRM, CRN, CRR, CRT, CRY, HKG, HKK, HKN, HKR, HRG, HRI, HRK, HRN, HRR, HRT, HRY, LKG, LKK, LKN, LKR, LKT, LKY, LRF, LRG, LRI, LRK, LRN, LRR, LRT, LRW, LRY, VKG, VKK, VKN, VKR, VKT, VKY, VRF, VRG, VRI, VRK, VRN, VRR, VRT, VRW, VRY
50	FFA, FMA, FML, FNA, FWA, FWL, FYA, FYL, FYV, GFA, GKA, GMA, GML, GMV, GNA, GWA, GWL, GYA, GYC, GYL, GYV, IFA, IMA, IML, INA, IWA, IYA, IYL, IYV, KFA, KMA, KMC, KML, KMV, KNA, KWA, KWL, KWV, KYA, KYC, KYH, KYL, KYV, MFA, MMA, MNA, MWA, MWL, MYA, MYL, MYV, NFA, NMA, NML, NMV, NNA, NWA, NWL, NWV, NYA, NYC, NYH, NYL, NYV, RFA, RMA, RML, RMV, RNA, RWA, RWL, RWV, RYA, RYC, RYH, RYL, RYV, TFA, TMA, TML, TNA, TWA, TWL, TYA, TYL, TYV, WMA, WWA, YFA, YKA, YMA, YML, YMV, YNA, YWA, YWL, YWV, YYA, YYH, YYL, YYV
51	CDF, CDI, CDK, CDM, CDN, CDR, CDT, CDY, CQF, CQI, CQK, CQM, CQN, CQR, CQT, CQY, DDF, DDG, DDI, DDK, DDM, DDN, DDR, DDT, DDY, DQF, DQI, DQM, DQT, DQY, HDE, HDF, HDG, HDI, HDK, HDM, HDN, HDR, HDT, HDW, HDY, HPF, HPI, HPK, HPM, HPN, HPR, HPT, HQE, HQF, HQG, HQI, HQK, HQM, HQN, HQR, HQT, HQW, HQY, HSF, HSI, HSK, HSM, HSN, HST, HSY, LDE, LDM, LQM, PDF, PDG, PDI, PDK, PDM, PDN, PDR, PDT, PDW, PDY, PQG, PQK, PQN, PQR, PQT, PQY, PSI, PSN, PST, QDE, QDF, QDG, QDI, QDK, QDM, QDN, QDR, QDT, QDW, QDY, QPF, QPI, QPT, QQF, QQG, QQI, QQK, QQM, QQN, QQR, QQT, QQW, QQY, QSF, QSG, QSI, QSM, QSN, QST, QSW, QSY
52	KGD, KGH, KGQ, KNH, KTH, KTQ, NGD, RGD, RGH, RGQ, RNH, RTH
53	EAE, EAK, ELE, EVE, FAA, FAE, FAK, FCE, FEE, FHE, FLA, FLE, FLV, FVA, FVE, IAE, ILE, IVE, KAE, KCE, KHE, KLE, KVE, MAA, MAE, MAF, MAK, MCE, MEE, MHE, MLA, MLE, MLK, MLL, MLV, MPE, MVA, MVE, MVK, NAE, NLE, NVE, RAE, RLE, RVE, TAE, WAE, WCE, WEE, WHE, WLE, WVE, YAA, YAC, YAE, YAF, YAI, YAK, YAL, YAM, YAV, YAW, YCA, YCE, YEE, YHE, YLA, YLE, YLH, YLK, YLL, YLV, YLW, YPE, YQE, YVA, YVE, YVK, YVL, YVV, YVW
54	DGS, DIS, DKS, DNS, DRS, DTS, DYS, QFS, QGD, QGP, QGS, QIS, QKS, QNP, QNS, QRS, QTS, QYS, SGS
55	PCA, PCH, PCL, PCV, PEA, PEL, PEV, PFA, PFC, PFH, PFL, PFP, PFV, PGA, PGC, PGH, PGL, PGP, PGQ, PGV, PGW, PIA, PIC, PIH, PIL, PIP, PIV, PKA, PKC, PKH, PKL, PKV, PMA, PMC, PMH, PML, PMV, PNA, PNC, PNH, PNL, PNP, PNV, PRA, PRC, PRH, PRL, PRV, PTA, PTC, PTH, PTL, PTP, PTV, PWA, PWC, PWH, PWL, PWV, PYA, PYC, PYH, PYL, PYP, PYV, QGH
56	FDD, FDP, FDQ, FDS, FPD, FPP, FPQ, FPS, FQD, FQP, FQQ, FQS, FSD, FSQ, GDD, GDP, GDQ, GDS, GPD, GPP, GPQ, GPS, GQD, GQP, GQQ, GQS, GSD, GSP, GSQ, GSS, IDD, IDP, IDQ, IDS, IPD, IPP, IPQ, IPS, IQD, IQP, IQQ, IQS, ISD, ISP, ISQ, ISS, KDD, KDP, KDQ, KDS, KPD, KPP, KPQ, KPS, KQD, KQP, KQQ, KQS, KSD, KSP, KSQ, KSS, MDD, MDP, MDQ, MDS, MPD, MPP, MPQ, MPS, MQD, MQP, MQQ, MQS, MSD, MSP, MSQ, MSS, NDD, NDP, NDQ, NDS, NPD, NPP, NPQ, NPS, NQD, NQP, NQQ, NQS, NSD, NSP, NSQ, NSS, RDD, RDP, RDQ, RDS, RPD, RPP, RPQ, RPS, RQD, RQP, RQQ, RQS, RSD, RSP, RSQ, RSS, TDD, TDP, TDQ, TDS, TPD, TPP, TPQ, TPS, TQD, TQP, TQQ, TQS, TSD, TSP, TSQ, TSS, WDD, WDS, WPD, WQS, WSD, YDD, YDP, YDQ, YDS, YPD, YPP, YPQ, YPS, YQD, YQP, YQQ, YQS, YSD, YSP, YSQ, YSS
57	CLI, CVI, HLI, HVI, PAA, PAC, PAE, PAF, PAG, PAI, PAK, PAM, PAN, PAR, PAT, PAV, PAW, PAY, PCC, PCE, PCF, PCI, PCK, PCM, PCN, PCT, PCW, PCY, PHC, PHE, PHF, PHI, PHK, PHM, PHN, PHT, PHV, PHW, PHY, PLA, PLC, PLE, PLF, PLG, PLH, PLI, PLK, PLM, PLN, PLR, PLT, PLV, PLW, PLY, PPC, PPF, PPI, PPM, PPT, PPW, PPY, PQF, PQI, PQM, PQW, PVA, PVC, PVE, PVF, PVG, PVH, PVI, PVK, PVM, PVN, PVR, PVT, PVV, PVW, PVY, VLF, VLM, VVF, VVM
58	SFE, SGE, SIE, SKE, SME, SNE, STE, SWE, SYE, SYK
59	EAD, EAP, EAQ, EAS, ECD, ECP, ECQ, EFD, EFQ, EFS, EGD, EGP, EGQ, EGS, EHD, EHP, EHQ, EID, EIQ, EIS, EKD, EKQ, EKS, EMD, EMQ, EMS, END, ENP, ENQ, ENS, EPD, EPP, EPS, ERD, ERQ, ERS, ETD, ETP, ETQ, ETS, EVD, EVP, EVQ, EVS, EWD, EWQ, EYD, EYQ, EYS
60	AAP, ALP, AVP, DAC, DAP, DCP, DHP, DLC, DLP, DVC, DVP, HAP, HCP, HFP, HHP, HIP, HLP, HVP, LAP, LLP, LVP, PAP, PAQ, PCP, PEP, PHP, PLP, PVP, QAC, QAP, QAV, QCP, QFP, QHP, QIP, QKP, QLC, QLH, QLP, QLQ, QPP, QVC, QVP, QWP, SAP, SCP, SLP, SVP, VAP, VLP, VVP
61	DAD, DAS, DCD, DCS, DHD, DHS, DLD, DLQ, DLS, DPD, DPQ, DPS, DQD, DQS, DVD, DVS, PLD, PLQ, PPD, PPS, PVD, PVQ, QAD, QCD, QCS, QHD, QHS, QLD, QLS, QPD, QPQ, QPS, QVD, QVS, SAD, SAQ, SAS, SCD, SCQ, SCS, SHD, SHQ, SHS, SLD, SLQ, SLS, SPD, SPQ, SPS, SQD, SQS, SVD, SVQ, SVS
62	EQL, FDA, FDL, FHA, FHL, FHV, FLL, FPA, FPL, FQA, FQC, FQH, FQL, FQV, GCA, GCV, GDA, GDC, GDH, GDL, GDV, GHA, GHL, GHV, GLL, GPA, GPH, GPL, GPV, GQA, GQC, GQH, GQL, GQV, GSL, IDA, IDC, IDH, IDL, IDV, IHA, IHL, IHV, ILL, IPA, IPL, IPV, IQA, IQC, IQH, IQL, IQV, KDA, KDC, KDH, KDL, KDV, KHA, KHL, KHV, KPA, KPL, KPV, KQA, KQC, KQH, KQL, KQV, MDA, MDL, MDV, MHA, MHL, MPA, MPL, MPV, MQA, MQC, MQH, MQL, MQV, NDA, NDC, NDH, NDL, NDV, NHA, NHL, NHV, NLL, NPA, NPL, NPV, NQA, NQC, NQH, NQL, NQV, RDA, RDC, RDH, RDL, RDV, RHA, RHL, RHV, RPA, RPL, RPV, RQA, RQC, RQH, RQL, RQV, RSL, TCA, TDA, TDC, TDH, TDL, TDV, THA, THL, THV, TLL, TPA, TPL, TPV, TQA, TQC, TQH, TQL, TQV, TSL, WHA, WHL, YDA, YDC, YDH, YDL, YDV, YHA, YHL, YHV, YPA, YPL, YPV, YQA, YQC, YQH, YQL, YQV, YSL
63	GGS, GIS, GNS, GRS, GTS, GYS, IGS, NGS, TGS, YGS
64	FED, FEH, FEP, FEQ, FKQ, IEH, IEQ, MED, MEH, MEP, MEQ, MHD, MKQ, MMQ, MWD, NEH, TEH, WED, WEH, WEP, WEQ, YED, YEH, YEP, YEQ, YES, YFD, YFQ, YHD, YHQ, YKD, YKH, YKQ, YMD, YMP, YMQ, YWD
65	AEV, CEA, CEL, CEV, EEC, EEV, FAL, FAV, FEA, FEC, FEL, FEV, FMV, FVL, FVV, FWV, GEA, GEC, GEL, GEV, GKV, GWV, HEA, HEV, IAL, IAV, ICV, IEA, IEC, IEE, IEL, IEP, IEV, IFV, IKA, IKL, IKV, ILV, IMV, IVL, IVV, IWL, IWV, KEA, KEC, KEL, KEV, MAL, MAV, MCV, MEA, MEC, MEL, MEV, MHV, MMV, MVL, MVV, MWV, NAV, NEA, NEC, NEL, NEV, NVV, REA, REC, REL, REV, RKV, TAL, TAV, TCV, TEA, TEC, TEL, TEV, TFV, TKA, TKL, TKV, TMV, TVL, TVV, TWV, VEV, WEA, WEL, WEV, YEA, YEC, YEL, YEV, YKV
66	DAF, DAI, DAM, DAN, DAW, DAY, DCY, DHM, DHY, DLF, DLI, DLM, DLN, DLT, DLW, DLY, DVF, DVI, DVM, DVN, DVT, DVW, DVY, HAY, HCY, HLN, HLT, HLY, HPY, HVN, HVT, HVY, QAF, QAG, QAI, QAK, QAM, QAN, QAR, QAT, QAW, QAY, QCK, QCN, QCY, QHF, QHI, QHM, QHN, QHW, QHY, QLF, QLG, QLI, QLK, QLM, QLN, QLR, QLT, QLW, QLY, QPM, QPY, QVF, QVG, QVI, QVK, QVM, QVN, QVR, QVT, QVW, QVY
67	ADE, ADF, ADG, ADI, ADK, ADM, ADN, ADR, ADT, ADW, ADY, APF, APG, API, APK, APN, APR, APT, APY, AQG, AQI, AQK, AQN, AQR, AQT, AQY, ASE, ASF, ASG, ASI, ASK, ASM, ASN, ASR, AST, ASW, ASY, CDG, CQG, CSG, CSN, CSR, CST, CSY, ESG, ESR, FSG, HPG, HSG, HSR, LDF, LDG, LDI, LDK, LDN, LDR, LDT, LDW, LDY, LPF, LPG, LPI, LPK, LPM, LPN, LPR, LPT, LPY, LQF, LQG, LQI, LQK, LQN, LQR, LQT, LQW, LQY, LSF, LSG, LSI, LSK, LSM, LSN, LSR, LST, LSW, LSY, PSG, VDF, VDG, VDI, VDK, VDM, VDN, VDR, VDT, VDW, VDY, VPF, VPG, VPI, VPK, VPN, VPR, VPT, VPY, VQG, VQI, VQK, VQN, VQR, VQT, VQY, VSF, VSG, VSI, VSK, VSM, VSN, VSR, VST, VSW, VSY, WDG, WSG, WSR, WSY

Continued on next page

Table 2 – continued from previous page

Codeword	Triplets
68	EFF, EFG, EFI, EFK, EFM, EFN, EFR, EFT, EFY, EGF, EGG, EGI, EGK, EGM, EGN, EGR, EGT, EGY, EIF, EIG, EII, EIK, EIM, EIN, EIR, EIT, EIY, EKF, EKG, EKI, EKK, EKM, EKN, EKR, EKT, EKY, EMF, EMG, EMI, EMK, EMN, EMR, EMT, EMY, ENF, ENG, ENI, ENK, ENM, ENN, ENR, ENT, ENY, ERF, ERG, ERI, ERK, ERM, ERN, ERR, ERT, ERY, ETF, ETG, ETI, ETK, ETM, ETN, ETR, ETT, ETY, EYF, EYG, EYI, EYK, EYM, EYN, EYR, EYT, EYY, FFK, FFN, FFT, FFY, FGG, FGI, FGK, FGN, FGR, FGT, FGY, FIG, FIK, FIN, FIR, FIT, FIY, FMY, FNG, FNK, FNN, FNR, FNT, FNY, FTG, FTK, FTN, FTR, FTT, FTY, FYG, FYK, FYN, FYR, FYT, FYY, ITT, KFN, KGF, KGI, KGT, KIN, KIR, KIY, KTN, KTR, KTT, KTY, KYN, KYR, MFK, MFN, MFR, MFT, MFY, MGG, MGI, MGK, MGN, MGR, MGT, MGY, MIG, MIK, MIN, MIR, MIT, MIY, MMK, MMN, MNG, MNK, MNN, MNR, MNT, MNY, MRK, MTG, MTK, MTN, MTR, MTT, MTY, MYG, MYK, MYN, MYR, MYT, MYY, WFG, WFI, WFK, WFN, WFR, WFT, WFY, WGF, WGG, WGI, WGK, WGM, WGN, WGR, WGT, WGY, WIG, WII, WIK, WIN, WIR, WIT, WIY, WKF, WKG, WKI, WKK, WKM, WKN, WKR, WKT, WKY, WMG, WMI, WMK, WMN, WMR, WMT, WMY, WNF, WNG, WNI, WNK, WNM, WNN, WNR, WNT, WNY, WRF, WRG, WRI, WRK, WRM, WRN, WRR, WRT, WRY, WTF, WTG, WTI, WTK, WTN, WTR, WTT, WTY, WYF, WYG, WYI, WYK, WYN, WYR, WYT, WYY
69	EAY, FAF, FAI, FAM, FAY, FCY, FLF, FLI, FLM, FLN, FLT, FLY, FVF, FVI, FVM, FVN, FVT, FVY, GCY, GHY, GLM, GLY, GVY, IAF, IAI, IAM, IAY, ICM, ICY, IHY, ILF, ILI, ILM, ILN, ILT, ILY, IVF, IVI, IVM, IVN, IVT, IVY, MAM, MAN, MAT, MAY, MCY, MLM, MLN, MLR, MLT, MLY, MVM, MVN, MVR, MVT, MVY, NAY, NLY, NVY, TAF, TAM, TAW, TAY, TCM, TCY, THM, THY, TLF, TLI, TLM, TLW, TLY, TVF, TVM, TVW, TVY, WLY, WVY, YAY, YCY, YLM, YLN, YLT, YLY, YVM, YVY
70	EAA, EAL, EAV, ECA, ECL, ECV, EEA, EEL, EFA, EFC, EFH, EFL, EFP, EFV, EGA, EGC, EGH, EGL, EGV, EHL, EIA, EIC, EIH, EIL, EIP, EIV, ELA, ELL, ELV, EMA, EMC, EMH, EML, EMP, EMV, ENA, ENC, ENH, ENL, ENV, ETA, ETC, ETH, ETL, ETV, EVA, EVL, EVV, EWA, EWC, EWH, EWL, EWV, EYA, EYC, EYH, EYL, EYP, EYV, MFL, MFV, MIA, MIL, MIV, MML, MTL
71	AAE, ALE, AVE, GAA, GAE, GAL, GAM, GAV, GAW, GCE, GCM, GEE, GHE, GLA, GLE, GLV, GLW, GPE, GQE, GVA, GVE, GVL, GVV, GVW, IAA, ICE, IHE, ILA, IVA, NAA, NCE, NHE, NLA, NLV, NVA, RCE, RHE, TAA, TCE, THE, TLA, TLE, TLV, TVA, TVE
72	APM, APW, AQE, AQF, AQM, AQW, EAF, EAI, EAM, EAN, EAT, ECC, ECE, ECF, ECI, ECK, ECM, ECN, ECT, ECW, ECY, EDC, EDE, EDF, EDG, EDI, EDK, EDM, EDN, EDR, EDT, EDW, EDY, EHA, EHC, EHE, EHF, EHG, EHH, EHI, EHK, EHM, EHN, EHR, EHT, EHV, EHW, EHY, ELC, ELF, ELG, ELI, ELK, ELM, ELN, ELR, ELT, ELW, ELY, EPC, EPE, EPF, EPG, EPH, EPI, EPK, EPM, EPN, EPR, EPT, EPW, EPY, EQA, EQC, EQE, EQF, EQG, EQH, EQI, EQK, EQM, EQN, EQR, EQT, EQV, EQW, EQY, ESE, ESF, ESI, ESK, ESM, ESN, EST, ESW, ESY, EVC, EVF, EVI, EVK, EVM, EVN, EVR, EVT, EVW, EVY, EWM, FHF, FHM, FQE, FQF, FQI, FQM, FQW, IHM, KHY, KPM, KQE, KQF, KQI, KQM, KQW, MCM, MDF, MDM, MDW, MHF, MHI, MHM, MPM, MQE, MQF, MQI, MQM, MQW, NHM, VPM, VQF, VQM, VQW, WDF, WDI, WDM, WHF, WHM, WPF, WPM, WQF, WQI, WQK, WQM, WQN, WQT, WQW, WQY
73	ACD, ACP, ACQ, ACS, AFS, AHD, AHP, AHS, AMS, AWD, AWP, AWQ, AWS, CWD, CWP, CWS, ECS, EHS, EWP, EWS, FCS, FHS, FWD, FWS, HWD, HWP, HWQ, HWS, ICS, IHS, IWS, KHS, KWS, LCD, LCP, LCQ, LCS, LFS, LHD, LHP, LHS, LMS, LWD, LWP, LWQ, LWS, VCD, VCP, VCQ, VCS, VFS, VHS, VMS, VWD, VWP, VWQ, VWS, WWS
74	GFF, GFI, GFM, GFN, GFT, GFY, GII, GIT, GKI, GKR, GKT, GKY, GMF, GMI, GMM, GMN, GMT, GMY, GNF, GNG, GNI, GNK, GNM, GNN, GNR, GNT, GNY, GTI, GTT, GWM, GYF, GYI, GYM, GYN, GYR, GYT, GYY, IFY, IKI, IKY, IMY, INI, INK, INN, INR, INT, INY, IYN, IYR, IYT, IYY, KFT, KFY, KKF, KKM, KKY, KMT, KMY, KNF, KNI, KNM, KNN, KNR, KNT, KNY, KYT, KYY, NFT, NFY, NKI, NKY, NMT, NMY, NNI, NNK, NNN, NNR, NNT, NNY, NYN, NYR, NYT, NYY, RFN, RFT, RFY, RKF, RKI, RKM, RKY, RMN, RMT, RMY, RNF, RNI, RNK, RNM, RNN, RNR, RNT, RNY, RYI, RYN, RYR, RYT, RYY, TFN, TFT, TFY, TKI, TKY, TMN, TMT, TMY, TNF, TNI, TNK, TNM, TNN, TNR, TNT, TNY, TYN, TYR, TYT, TYY, YNK, YNN, YNR, YNT, YYR
75	DAE, DCE, DLE, DVE, QAE, QLE, QVE, SAE, SAF, SAM, SAW, SCE, SEE, SLE, SLM, SLW, SVE, SVM, SVW
76	DAG, DAK, DAR, DAT, DLG, DLK, DLR, DVG, DVK, DVR, SAG, SAI, SAK, SAN, SAR, SAT, SAY, SCI, SCR, SCT, SLF, SLG, SLI, SLK, SLN, SLR, SLT, SLY, SVF, SVG, SVI, SVK, SVN, SVR, SVT, SVY
77	DEF, DEG, DEI, DEK, DEM, DEN, DER, DET, DEW, DEY, HEG, HEK, HEN, HER, HET, HEY, PEF, PEG, PEI, PEK, PEM, PEN, PER, PET, PEY, QEA, QEF, QEG, QEI, QEK, QEN, QER, QET, QEY, QMR, SEF, SEG, SEI, SEK, SEM, SEN, SER, SET, SEW, SEY
78	DKE, DKF, DKK, DKM, DKW, DRE, DRF, DRH, DRI, DRK, DRM, DRW, PRE, QKE, QKW, QRE, QRF, QRH, QRK, QRM, QRW, SRE
79	DKD, DKQ, DRD, DRP, DRQ, QKQ, QRD, QRP, QRQ, SKD, SKP, SKQ, SKS, SRD, SRH, SRP, SRQ, SRS
80	CFP, CGP, CIP, CNP, CTP, CYP, FFP, FGP, FIP, FKP, FNP, FRP, FTP, FYP, GGP, GIP, GKP, GNP, GRP, GTP, GYP, IFP, IGP, IIP, IKP, INP, IRP, ITP, IYP, KCP, KFP, KGC, KGP, KHP, KIP, KKP, KMP, KNP, KRP, KTP, KWP, KYP, MCP, MFP, MGP, MIP, MKP, MMP, MNP, MRP, MTP, MYP, NCP, NFP, NGC, NGP, NIP, NKP, NMP, NNP, NRP, NTP, NYP, RCP, RFP, RGC, RGP, RIP, RKP, RNP, RRP, RTP, RWP, RYP, TFP, TGP, TIP, TKP, TNP, TRP, TTP, TYP, YFP, YGP, YIP, YKP, YNP, YRP, YTP, YYP
81	KAA, KAC, KAF, KAG, KAI, KAK, KAM, KAN, KAR, KAT, KAV, KAW, KAY, KCA, KCC, KCF, KCI, KCK, KCM, KCN, KCT, KCV, KCW, KCY, KHC, KHF, KHI, KHK, KHM, KHW, KLA, KLC, KLF, KLG, KLH, KLI, KLK, KLL, KLM, KLN, KLR, KLT, KLV, KLW, KLY, KVA, KVC, KVF, KVG, KVI, KVK, KVL, KVM, KVN, KVR, KVT, KVV, KVW, KVY, MAI, MLF, MLI, MVF, MVI, NAC, NAF, NAI, NAM, NCF, NCM, NLC, NLF, NLI, NLM, NLW, NVC, NVF, NVI, NVM, NVW, RAA, RAC, RAF, RAG, RAI, RAK, RAM, RAN, RAR, RAT, RAV, RAW, RAY, RCA, RCC, RCF, RCI, RCK, RCM, RCN, RCR, RCT, RCV, RCW, RCY, RHC, RHF, RHI, RHK, RHM, RHN, RHT, RHW, RHY, RLA, RLC, RLF, RLG, RLH, RLI, RLK, RLL, RLM, RLN, RLR, RLT, RLV, RLW, RLY, RVA, RVC, RVF, RVG, RVI, RVK, RVM, RVN, RVR, RVT, RVV, RVW, RVY, RWM, TLC, TVI, YLC, YLF, YLI, YVC, YVF, YVI
82	FKK, FRK, GKE, GKF, GKK, GKM, GKN, GRC, GRE, GRF, GRH, GRI, GRK, GRM, GRN, GRR, GRT, GRW, GRY, IKK, IRK, IRN, KKK, KRK, KRM, NKF, NKK, NKM, NKN, NRF, NRK, NRM, NRN, RKK, RRF, RRK, RRM, TKF, TKK, TKM, TKN, TRE, TRF, TRK, TRM, TRN, YKK, YRK
83	AAD, AAQ, AEC, AED, AEH, AEL, AEP, AEQ, AES, AFH, AFP, AFQ, AHH, AIH, AMH, AMP, AMQ, AVD, AVQ, AWH, CED, CEH, CEP, CEQ, EED, EEH, EEP, EEQ, HED, HEH, HEL, HEP, HEQ, LAQ, LEC, LED, LEH, LEL, LEP, LEQ, LES, LEV, LFH, LFQ, LHH, LIH, LMH, LMP, LMQ, LVQ, LWH, VAQ, VEC, VED, VEH, VEL, VEP, VEQ, VES, VFH, VFQ, VMH, VMP, VMQ, VVQ, VWH
84	DHF, DHI, DHK, DHN, DHR, DPG, DPI, DPK, DPN, DPR, DPT, DPY, DQG, DQK, DQN, DQR, PPG, PPK, PPN, PPR, QHK, QHR, QPG, QPK, QPN, QPR, SCF, SCK, SCM, SCN, SCY, SDG, SDN, SHF, SHG, SHI, SHK, SHM, SHN, SHR, SHT, SHY, SPF, SPG, SPI, SPK, SPN, SPR, SPT, SPY, SQG, SQI, SQK, SQN, SQR, SQT, SQY
85	FFF, FFI, FFM, FGM, FIF, FII, FIM, FMF, FMI, FMM, FNI, FRM, FTF, FTI, FTM, FYF, FYI, FYM, GIF, GIM, GTF, GTM, IFF, IFI, IFM, IGI, IIF, III, IIM, IIY, IMF, IMI, IMM, ITF, ITI, ITM, IYI, IYM, KFF, KFI, KFM, KIF, KII, KIM, KMF, KMI, KMM, KTF, KTI, KTM, KYF, KYI, KYM, MEF, MEM, MFF, MFI, MFM, MGF, MGM, MIF, MII, MIM, MKF, MKI, MKM, MMF, MMI, MMM, MMY, MNF, MNI, MNM, MRF, MRI, MRM, MTF, MTI, MTM, MYF, MYI, MYM, NFF, NFI, NFM, NGF, NGI, NGM, NIF, NII, NIM, NMF, NMI, NMM, NNF, NNM, NTF, NTI, NTM, NYF, NYI, NYM, RFF, RFI, RFM, RIF, RII, RIM, RIW, RMF, RMI, RMM, RTF, RTI, RTM, RYF, RYM, TFF, TFI, TFM, TIF, TII, TIM, TMF, TMI, TMM, TTF, TTI, TTM, TYF, TYI, TYM, WFF, WFM, WIF, WIM, WMF, WMM, WTM, WYM, YCM, YEF, YEM, YEW, YFC, YFF, YFI, YFK, YFM, YFN, YFT, YFW, YFY, YGF, YGI, YGM, YIC, YIF, YII, YIK, YIM, YIN, YIT, YIW, YIY, YKF, YKI, YKM, YMC, YMF, YMI, YMK, YMM, YMN, YMT, YMW, YMY, YNF, YNI, YNM, YNY, YRF, YRI, YRM, YTC, YTF, YTI, YTK, YTM, YTN, YTT, YTW, YTY, YWM, YYC, YYF, YYI, YYM, YYN, YYT, YYW, YYY

Continued on next page

Table 2 – continued from previous page

Codeword	Triplets
86	DFA, DFL, DFV, DGA, DGC, DGL, DGV, DIA, DIL, DIV, DKA, DKL, DKV, DMA, DML, DMV, DNA, DNC, DNL, DNV, DRA, DRC, DRL, DRV, DTA, DTC, DTL, DTV, DWA, DWV, DYA, DYL, DYV, QFL, QGA, QGC, QGL, QGV, QIA, QIL, QIV, QKA, QKL, QKV, QML, QNA, QNL, QNV, QRA, QRC, QRL, QRV, QTA, QTC, QTL, QTV, QWL, QYA, QYL, QYV, SFA, SFL, SFV, SGA, SGL, SGV, SIA, SIL, SIV, SKA, SKC, SKL, SKV, SMA, SMC, SML, SMV, SNA, SNL, SNV, SRA, SRC, SRL, SRV, STA, STL, STV, SWA, SWL, SWV, SYA, SYC, SYL, SYV
87	ELD, ELP, ELQ, FAD, FAP, FAQ, FCD, FLD, FLP, FLQ, FVD, FVP, FVQ, GAD, GAP, GAQ, GAS, GCD, GCS, GHD, GHS, GLD, GLP, GLQ, GLS, GVD, GVP, GVQ, GVS, IAD, IAP, IAQ, IAS, ICD, IHD, ILD, ILP, ILQ, ILS, IVD, IVP, IVQ, IVS, KAD, KAP, KAQ, KAS, KCD, KHD, KLD, KLP, KLQ, KLS, KVD, KVP, KVQ, KVS, MAD, MAP, MAQ, MCD, MLD, MLP, MLQ, MLS, MVD, MVP, MVQ, MVS, NAD, NAP, NAQ, NAS, NCD, NCS, NHD, NHS, NLD, NLP, NLQ, NLS, NVD, NVP, NVQ, NVS, RAD, RAL, RAP, RAQ, RAS, RCD, RCQ, RHD, RHP, RHS, RLD, RLP, RLQ, RLS, RVD, RVL, RVP, RVQ, RVS, TAD, TAP, TAQ, TAS, TCD, TCS, THD, TLD, TLP, TLQ, TLS, TVD, TVP, TVQ, TVS, YAD, YAP, YAQ, YAS, YCD, YCS, YLD, YLP, YLQ, YLS, YVD, YVP, YVQ, YVS
88	AFD, AGD, AGH, AGQ, AID, AIQ, AKD, AKP, AKQ, AMD, AND, ANQ, ARD, ARP, ARQ, ATD, ATH, ATQ, AYD, AYQ, CRQ, CTQ, LFD, LGD, LGH, LGQ, LID, LIQ, LKD, LKP, LKQ, LMD, LND, LNQ, LRD, LRP, LRQ, LTD, LTH, LTQ, LYD, LYQ, VFD, VGD, VGH, VGQ, VID, VIQ, VKD, VKP, VKQ, VMD, VND, VNQ, VRD, VRP, VRQ, VTD, VTH, VTQ, VYD, VYQ
89	DSA, DSC, DSD, DSH, DSL, DSP, DSQ, DSS, DSV, PSP, QSC, QSH, QSL, QSQ, QSV, SSA, SSC, SSD, SSH, SSL, SSP, SSQ, SSS, SSV
90	FFE, FIE, FKE, FME, FMK, FWE, FYE, GFE, GFK, GGE, GIE, GME, GNE, GTE, GWE, GYE, GYK, IFE, IFK, IIE, IIK, IKE, IME, IMK, INE, IRE, ITE, IWE, IYE, IYK, KEE, KFE, KFK, KGE, KIE, KIK, KKE, KME, KMK, KNE, KNK, KRE, KTE, KWE, KYE, KYK, MFE, MIE, MKE, MME, MNE, MTE, MWE, MYE, NEE, NFE, NFK, NGE, NIE, NIK, NKE, NME, NNE, NRE, NTE, NWE, NYE, NYK, REE, RFE, RFK, RGE, RIE, RKE, RME, RMK, RNE, RRE, RTE, RWE, RYE, RYK, TEE, TFE, TFK, TGE, TIE, TKE, TME, TMK, TNE, TTE, TWE, TYE, TYK, YFE, YGE, YIE, YKE, YME, YNE, YRE, YTE, YWE, YYE, YYK
91	ADA, ADL, ADV, AQA, AQV, ASA, ASC, ASL, ASV, CDA, CDC, CDE, CDH, CDL, CDV, CDW, CPA, CPC, CPE, CPH, CPL, CPV, CQA, CQC, CQE, CQH, CQL, CQV, CSA, CSC, CSE, CSF, CSH, CSI, CSK, CSL, CSM, CSP, CSV, CSW, EDA, EDL, EDV, ESA, ESC, ESH, ESL, ESP, ESV, FDC, FDH, FDV, FPV, FSA, FSC, FSH, FSL, FSP, FSV, HDA, HDC, HDH, HDL, HDV, HPA, HPC, HPE, HPH, HPL, HPV, HQA, HQC, HQL, HQV, HSA, HSC, HSE, HSH, HSL, HSP, HSV, HSW, ISA, ISL, ISV, KSA, KSL, KSV, LDA, LDL, LDV, LQA, LQV, LSA, LSC, LSE, LSL, LSV, MDC, MDH, MSA, MSC, MSH, MSL, MSV, NSA, NSL, NSV, PDA, PPA, PPV, PSA, PSC, PSH, PSL, PSV, QSA, VDA, VDC, VDE, VDL, VDV, VQA, VQE, VQL, VQV, VSA, VSC, VSE, VSL, VSV, WDA, WDC, WDE, WDH, WDK, WDL, WDP, WDV, WDW, WHV, WLL, WPA, WPC, WPE, WPH, WPL, WPP, WPV, WQA, WQC, WQE, WQH, WQL, WQP, WQV, WSA, WSC, WSE, WSF, WSH, WSI, WSK, WSL, WSM, WSN, WSP, WSQ, WST, WSV, WSW
92	DNM, DTF, DTM, DYC, DYF, DYM, DYW, HGF, HGM, HNF, HNM, HNW, HRF, HRM, HRW, HTF, HTI, HTM, HTW, HYC, HYF, HYH, HYI, HYK, HYM, HYN, HYT, HYW, HYY, PYF, PYI, PYM, PYW, QGM, QGW, QNC, QNM, QNW, QTF, QTI, QTM, QTW, QYC, QYF, QYH, QYI, QYM, QYW, QYY, SYF, SYM
93	CFD, CFQ, CID, CIQ, CKD, CKP, CKQ, CKS, CMD, CMP, CMQ, CMS, CND, CNQ, CRD, CTD, CYD, CYQ, HFD, HFQ, HID, HIQ, HKD, HKP, HKQ, HKS, HMD, HMP, HMQ, HMS, HND, HNP, HNQ, HNS, HRD, HRQ, HRS, HTD, HTQ, HYD, HYP, HYQ, HYS, PCD, PCQ, PCS, PED, PEQ, PES, PFD, PFQ, PFS, PGD, PGS, PHD, PHQ, PHS, PID, PIQ, PIS, PKD, PKP, PKQ, PKS, PMD, PMP, PMQ, PMS, PND, PNQ, PNS, PRD, PRP, PRQ, PRS, PTD, PTQ, PTS, PWD, PWQ, PWS, PYD, PYQ, PYS, QKD, QND
94	AEA, AFA, AFL, AFV, AIA, AIL, AIV, AMA, AML, AMV, ANA, ATA, ATL, ATV, AWA, AWL, AWV, AYA, AYC, AYH, AYL, AYV, HMA, HML, HYA, LEA, LFA, LFL, LFV, LIA, LIL, LIV, LMA, LML, LMV, LNA, LTA, LTL, LTV, LWA, LWL, LWV, LYA, LYH, LYL, LYV, VEA, VFA, VFL, VFV, VIA, VIH, VIL, VIV, VMA, VML, VMV, VNA, VNL, VTA, VTL, VTV, VWA, VWL, VWV, VYA, VYH, VYL, VYV
95	CCA, CFA, CFL, CFV, CGA, CGC, CGF, CGH, CGI, CGL, CGV, CIA, CIL, CIV, CMA, CML, CMV, CNA, CNC, CNH, CNL, CNV, CTA, CTC, CTH, CTL, CTV, CVA, CWA, CWL, CWV, CYA, CYC, CYH, CYL, CYV, FGA, HFA, HFL, HFV, HGA, HGC, HGH, HGL, HGV, HIA, HIL, HIV, HNA, HNC, HNH, HNL, HNV, HTA, HTC, HTH, HTL, HTV, HWA, HWL, HYL, HYV, WAA, WAL, WCA, WCL, WCV, WFA, WFL, WFV, WGA, WGC, WGH, WGL, WGV, WIA, WIC, WIL, WIV, WKA, WML, WMV, WNA, WNL, WNV, WRA, WRL, WRV, WTA, WTC, WTH, WTL, WTV, WVA, WVL, WWL, WWV, WYA, WYL, WYV
96	KCS, KFS, KGS, KIS, KKS, KNS, KRS, KTS, KYS, RCS, RFS, RGS, RIS, RKS, RNS, RRS, RTS, RWS, RYS
97	FAG, FAN, FAR, FAT, FLG, FLK, FLR, FVG, FVK, FVR, GAF, GAG, GAI, GAK, GAN, GAR, GAT, GAY, GCK, GLC, GLF, GLG, GLI, GLK, GLN, GLR, GLT, GVF, GVG, GVI, GVK, GVM, GVN, GVR, GVT, IAG, IAK, IAN, IAR, IAT, ILG, ILK, ILR, IVG, IVK, IVR, MAG, MAR, MLG, MVG, NAG, NAK, NAN, NAR, NAT, NLG, NLK, NLN, NLR, NLT, NVG, NVK, NVN, NVR, NVT, TAG, TAI, TAK, TAN, TAR, TAT, TLG, TLK, TLN, TLR, TLT, TVG, TVK, TVN, TVR, TVT, WAR, WLR, WVR, WVT, YAG, YAN, YAR, YAT, YLG, YLR, YVG, YVN, YVR, YVT
98	AGW, CGW, CNW, CRW, CTW, EEW, EFW, EGW, EIW, EKW, EMW, ENW, ERW, ETW, EWW, EYW, FCC, FCW, FEW, FFC, FFW, FGC, FGF, FGW, FHC, FHW, FIC, FIW, FKC, FKF, FKH, FKM, FKW, FMC, FMW, FNC, FNF, FNH, FNM, FNW, FPW, FRC, FRF, FRH, FRW, FTC, FTH, FTW, FWC, FWW, FYC, FYH, FYW, GCW, GFW, GGC, GGW, GHW, GIW, GKC, GKW, GMW, GNC, GNW, GTC, GTW, GWW, GYW, ICC, ICW, IEW, IFC, IFW, IGC, IGF, IGM, IGW, IHW, IIC, IIW, IKC, IKF, IKH, IKM, IKW, IMC, IMW, INC, INF, INH, INM, INW, IRC, IRF, IRH, IRM, IRW, ITC, ITH, ITW, IWC, IWW, IYC, IYF, IYH, IYW, KFW, KGW, KIW, KKW, KMW, KNC, KNW, KRW, KTW, KWW, KYW, MCC, MCW, MEW, MFC, MFW, MGC, MGW, MHC, MHW, MIC, MIW, MKC, MKH, MKW, MMC, MMW, MNC, MNH, MNW, MPH, MPW, MRC, MRH, MRW, MTC, MTH, MTW, MWC, MWW, MYC, MYH, MYW, NCW, NEW, NFW, NGW, NHW, NIW, NKC, NKW, NMW, NNC, NNH, NNW, NRC, NRH, NRW, NTW, NWW, NYW, RFW, RGW, RKC, RKW, RMW, RNC, RNW, RRW, RTW, RWW, RYW, TCW, TEW, TFW, TGC, TGW, THW, TIC, TIW, TKC, TKW, TMW, TNC, TNH, TNW, TRC, TRH, TRW, TTC, TTW, TWW, TYC, TYH, TYW, WFC, WFW, WGW, WIW, WKC, WKH, WKW, WMC, WMW, WNC, WNH, WNW, WRC, WRW, WTW, WWC, WWW, WYC, WYH, WYW, YCW, YGC, YGW, YHW, YKC, YKW, YNC, YNH, YNW, YRC, YRH, YRW, YWW
99	ADC, ADD, ADH, ADP, ADQ, AHQ, ALD, ALQ, APD, APP, APQ, AQC, AQD, AQH, AQL, AQP, AQQ, AQS, ASH, ASQ, CDD, CDP, CDQ, CDS, CHQ, CPD, CPP, CPQ, CQD, CQP, CQQ, CQS, CSD, CSQ, EDD, EDH, EDP, EDQ, EPQ, EQD, EQP, EQQ, EQS, ESQ, HDD, HDP, HDQ, HDS, HHD, HHQ, HPD, HPP, HPQ, HQD, HQH, HQP, HQQ, HQS, HSD, HSQ, LDC, LDD, LDH, LDP, LDQ, LHQ, LLD, LLQ, LPD, LPH, LPP, LPQ, LQC, LQD, LQH, LQL, LQP, LQQ, LQS, LSD, LSH, LSP, LSQ, PDD, PDQ, PDS, PPQ, PQD, PQQ, PQS, PSD, PSQ, QDD, QQD, QSD, VDD, VDH, VDP, VDQ, VDS, VHD, VHH, VHP, VHQ, VLQ, VPD, VPH, VPP, VPQ, VQC, VQD, VQH, VQP, VQQ, VQS, VSD, VSH, VSP, VSQ, WDQ, WPQ, WQD, WQQ
100	DCF, DCM, DCW, DFC, DFF, DFI, DFM, DFN, DFW, DFY, DIC, DIF, DII, DIM, DIW, DIY, DMC, DMF, DMI, DMM, DMW, DMY, DWF, DWM, DWW, HCF, HCI, HCM, HEF, HEM, HFC, HFF, HFH, HFI, HFK, HFM, HFN, HFT, HFW, HFY, HIC, HIF, HII, HIK, HIM, HIN, HIT, HIW, HIY, HMC, HME, HMF, HMH, HMI, HMK, HMM, HMN, HMT, HMV, HMW, HMY, HWC, HWE, HWF, HWI, HWM, HWV, HWW, PFF, PFI, PFM, PFW, PIF, PII, PIM, PIW, PMF, PMI, PMM, PMW, PWF, PWI, PWM, PWW, QCC, QCF, QCI, QCM, QCW, QEM, QEW, QFA, QFC, QFF, QFI, QFK, QFM, QFN, QFT, QFV, QFW, QFY, QIC, QIF, QII, QIK, QIM, QIN, QIT, QIW, QIY, QMA, QMC, QMF, QMI, QMK, QMM, QMN, QMT, QMV, QMW, QMY, QWA, QWC, QWF, QWI, QWM, QWV, QWW, SFF, SFI, SFM, SIF, SIM, SMF, SMI, SMM, SWF, SWM

	Seqs[1]	TcR Seqs[2]	Functional[3]	Distinct nt[4]	Distinct CDR3[5]
U	1,788,551	428,326	404,032	186,599	113,429
U	3,430,471	873,607	831,605	303,266	171,004
U	1,023,799	172,105	164,114	97,601	69,125
U	2,394,189	175,924	166,398	74,141	48,244
U	2,949,666	347,621	326,310	106,112	65,806
U	3,840,471	216,790	207,135	38,902	27,660
D5	10,005,718	2,034,715	1,931,868	720,436	415,436
D5	2,770,773	930,044	884,619	408,830	256,116
D5	4,479,250	1,589,692	1,512,244	432,490	267,451
D5	2,480,475	834,436	795,269	205,329	138,134
D5	10,413,014	2,730,565	2,576,696	1,008,680	558,64
D5	10,578,666	2,050,542	1,946,156	531,214	317,637
D14	5,689,589	1,884,138	1,792,945	570,080	346,164
D14	4,297,876	1,320,231	1,254,062	517,549	323,291
D14	6,415,997	1,260,362	1,205,562	371,107	236,863
D14	4,251,640	1,258,960	1,203,263	372,241	236,572
D14	4,220,098	842,190	799,261	318,764	206,532
D14	5,829,487	1,182,640	1,131,125	413,942	260,594
M2	4,497,142	424,287	406,912	154,001	107,337
M2	6,570,454	441,799	422,609	111,140	82,213
M2	6,754,251	764,024	732,688	153,383	110,533
M2	5,303,993	357,340	341,430	110,816	83,392
M2	3,601,458	339,273	321,471	203,614	139,381
M2	7,124,801	410,596	387,232	177,126	121,348

Table 1: Summary of murine HTS TcR data used in this study. The analysis described in this study focused exclusively on functional TcRs. (1) The number of sequences generated which pass the sequence quality threshold. (2) The number of sequences recognized and classified by Decombinator as TcRs. (3) The number of sequences containing an in frame CDR3.(4) The number of TcRs obtained counting each distinct sequence once only. (5) The number of CDR3 sequences obtained, counting each CDR3 sequence once only.
